# The molecular characteristics of high-grade gastroenteropancreatic neuroendocrine neoplasms

**DOI:** 10.1530/ERC-21-0152

**Published:** 2021-10-14

**Authors:** Andreas Venizelos, Hege Elvebakken, Aurel Perren, Oleksii Nikolaienko, Wei Deng, Inger Marie B Lothe, Anne Couvelard, Geir Olav Hjortland, Anna Sundlöv, Johanna Svensson, Harrish Garresori, Christian Kersten, Eva Hofsli, Sönke Detlefsen, Merete Krogh, Halfdan Sorbye, Stian Knappskog

**Affiliations:** 1 K.G. Jebsen Center for Genome-Directed Cancer Therapy, Department of Clinical Science, University of Bergen, Bergen, Norway; 2 Department of Oncology, Haukeland University Hospital, Bergen, Norway; 3 Department of Oncology, Ålesund Hospital, Møre og Romsdal Hospital Trust, Ålesund, Norway; 4 Department of Clinical and Molecular Medicine, Faculty of Medicine and Health Sciences, Norwegian University of Science and Technology, Trondheim, Norway; 5 Institute of Pathology, University of Bern, Bern, Switzerland; 6 Department of Pathology, Oslo University Hospital, Oslo, Norway; 7 Department of Pathology, Université de Paris, Bichat Hospital, AP-HP, Paris, France; 8 Department of Oncology, Oslo University Hospital, Oslo, Norway; 9 Departmentt of Oncology, Skåne University Hospital, Lund, Sweden; 10 Department of Medical Radiation Physics, Lund University, Lund, Sweden; 11 Department of Oncology, Sahlgrenska University Hospital, Gothenburg, Sweden; 12 Department of Oncology, Stavanger University Hospital, Stavanger, Norway; 13 Department of Research, Hospital of Southern Norway, Kristiansand, Norway; 14 Department of Oncology, St.Olavs Hospital, Trondheim, Norway; 15 Department of Pathology, Odense University Hospital, Odense, Denmark; 16 Department of Clinical Medicine, Faculty of Health Sciences, University of Southern Denmark, Odense, Denmark; 17 Department of Oncology, Odense University Hospital, Odense, Denmark; 18 Department of Clinical Science, University of Bergen, Bergen, Norway

**Keywords:** neuroendocrine neoplasms, neuroendocrine carcinoma, high-grade, gastroenteropancreatic, genetic alterations, molecular markers

## Abstract

High-grade (HG) gastroenteropancreatic (GEP) neuroendocrine neoplasms (NEN) are rare but have a very poor prognosis and represent a severely understudied class of tumours. Molecular data for HG GEP-NEN are limited, and treatment strategies for the carcinoma subgroup (HG GEP-NEC) are extrapolated from small-cell lung cancer (SCLC). After pathological re-evaluation, we analysed DNA from tumours and matched blood samples from 181 HG GEP-NEN patients; 152 neuroendocrine carcinomas (NEC) and 29 neuroendocrine tumours (NET G3). Based on the sequencing of 360 cancer-related genes, we assessed mutations and copy number alterations (CNA). For NEC, frequently mutated genes were *TP53* (64%), *APC* (28%), *KRAS* (22%) and *BRAF* (20%). *RB1* was only mutated in 14%, but CNAs affecting *RB1* were seen in 34%. Other frequent copy number losses were *ARID1A* (35%), *ESR1* (25%) and *ATM* (31%). Frequent amplifications/gains were found in *MYC* (51%) and *KDM5A* (45%). While these molecular features had limited similarities with SCLC, we found potentially targetable alterations in 66% of the NEC samples. Mutations and CNA varied according to primary tumour site with *BRAF* mutations mainly seen in colon (49%), and *FBXW7* mutations mainly seen in rectal cancers (25%). Eight out of 152 (5.3%) NEC were microsatellite instable (MSI). NET G3 had frequent mutations in *MEN1* (21%), *ATRX* (17%), *DAXX, SETD2* and *TP53* (each 14%). We show molecular differences in HG GEP-NEN, related to morphological differentiation and site of origin. Limited similarities to SCLC and a high fraction of targetable alterations indicate a high potential for better-personalized treatments.

## Introduction

High-grade (HG) gastroenteropancreatic (GEP) neuroendocrine neoplasms (NEN) are defined by the presence of neuroendocrine phenotype and a high proliferation rate (Ki-67 > 20%). The HG NEN entity consists of well-differentiated neuroendocrine tumours (NET G3) and poorly differentiated neuroendocrine carcinoma (NEC) (WHO 2019). GEP-NEC have a particularly unfavourable prognosis, with median overall survival <1 year in advanced, treated cases and only 1 month if untreated (Sorbye *et al.* 2013, Yamaguchi *et al.* 2014, Heetfeld *et al.* 2015, Walter *et al.* 2017). Currently, the molecular mechanisms behind this aggressive phenotype remain unknown.

Among the HG NEN patients, those with GEP-NET G3 have better survival compared to NEC, but an inferior response to platinum/etoposide-based chemotherapy (Heetfeld *et al.* 2015). The treatment strategy (platinum/etoposide chemotherapy) for adjuvant and metastatic GEP-NEC has been extrapolated from small-cell lung cancer (SCLC) (Strosberg *et al.* 2010, Garcia-Carbonero *et al.* 2016), based on clinical, morphological and immunohistochemical similarities. However, previous studies have revealed clinical differences between SCLC and GEP-NEC, questioning this approach (Brennan *et al.* 2010, Dasari *et al.* 2018).

Regarding potential biomarkers, the benefit of platinum-based treatment for pancreatic NEN G3 has been reported to depend on *KRAS* mutations and loss of *RB1* (Hijioka *et al.* 2017) and studies on NEC have associated microsatellite instability (MSI) with improved prognosis (La Rosa *et al.* 2012, Sahnane *et al.* 2015). However, molecular markers for classification, treatment selection and prognosis for HG GEP-NEN are generally lacking. Some molecular features of GEP-NEC with primary sites in the colon, rectum and pancreas have similarities to their adenocarcinoma counterparts of the same organs (Takizawa *et al.* 2015, Konukiewitz *et al.* 2018). This could indicate a shared genetic origin, but so far this has not been taken into account in the choice of medical treatment.

The 2019 WHO classification of digestive tumours state that NEC frequently have mutations in *TP53* and *RB1*, whereas pancreatic NET G3 retain the mutation profile of other well-differentiated NET (WHO 2019). More detailed assessments report *TP53*, *KRAS*, *BRAF* and *PIK3CA/PTEN* as the most frequently mutated genes in GEP-NEC, but studies and number of cases are limited (Vijayvergia *et al.* 2016, Busico *et al.* 2020). *MEN1*, *DAXX* and *ATRX* are the most frequently mutated genes in pancreatic G1-G2 NET (Ki-67 < 20%) (Jiao *et al.* 2011), and similar alterations are reported in pancreatic NET G3 (Yachida *et al.* 2012, Tang *et al.* 2016*a*, Konukiewitz *et al.* 2018). Mutations in NET G3 outside of the pancreas seem to be rare (Busico *et al.* 2020). These findings indicate that NET G3 and NEC belong to two different types of malignancies, also on the molecular level, and that molecular characteristics may be used to differentiate and classify NEC from NET G3 in cases where morphology is not sufficient (Tang *et al.* 2016*a*,*b*).

In this study, after pathological re-evaluation, we performed massive parallel sequencing of a panel of 360 cancer-related genes across a large set of GEP-NEC (*n* = 152) and NET G3 samples (*n* = 29), all with matched normal tissue (blood). We provide an overview of the molecular landscape in high-grade GEP-NEN and thereby pave the way for a better understanding of the molecular mechanisms and genetic origin of these tumours as well as why these cancers are so aggressive. Importantly, we reveal a high fraction of targetable alterations in HG GEP-NEN patients, pointing to novel treatment strategies applying tailored therapies.

## Materials and methods

### Study design

The goal of this study was to perform an extensive molecular characterization of HG GEP-NEN and thereby to provide a basis for improved diagnostic accuracy, prognosis estimation and development of possible new treatment strategies. For this purpose, we applied massive parallel sequencing (NGS) with subsequent assessments of genetic alterations, in a large biobank of HG GEP-NEN samples.

### Patients and samples

The samples were from patients diagnosed with HG GEP-NEN during 2013–2017 that had been prospectively included in a Nordic registry. Inclusion criteria were histopathologically confirmed high-grade neuroendocrine neoplasm (Ki-67 > 20%) with gastroenteropancreatic primary or unknown primary site (CUP) with predominantly gastrointestinal metastases (defined by radiological CT scans). Clinical information, tumour tissue and a whole blood sample for normal tissue analyses were collected for each case. Thus, 181 cases were finally included for the present analyses (see Supplementary Methods for details, see section on supplementary materials given at the end of this article). Histological sections (HE, CgA, synaptophysin, Ki-67) were collected, digitalized and subjected to a centralized pathological re-evaluation (A P) for validation of HG NEN diagnosis, WHO 2019 classification, cell-type and Ki-67 recount. Thereafter, an additional blinded pathology review was done by three pathologists (A P, A C and I M B L) for all cases (*n* = 68) meeting the following criteria: NET G3 or non-small cell NEC with a Ki-67 ≤ 55% or uncertain morphology (*n* = 9), since these are the cases where pathology assessment separating NET G3 from NEC is important. Difficult cases were finally discussed during a virtual consensus meeting. Among the 181 cases, 152 were classified as NEC and 29 as NET G3, reflecting an expected distribution between NEC and NET G3.

### Tissue collection and isolation of DNA

For tumour samples, tissue cores from areas with high tumour cell content were collected, and DNA was isolated using ultrasonication and subsequent column-based binding and elution (Supplementary Methods). Genomic DNA from normal tissue (blood) was isolated using QIAamp DNA MiniKit (Qiagen). MSI status was determined using the Promega MSI analysis system (Version1.2, Promega).

### Library preparation and sequencing

Subsequent to DNA quality controls (Supplementary Methods), targeted massive parallel sequencing was performed on DNA from FFPE tumour tissue and from matched normal peripheral blood DNA. Illumina libraries were prepared applying Kapa Hyper Prep kit (Kapa Biosystem) and Agilent SureSelect XT-kit (Agilent). Targeted enrichment was performed using RNA baits (SureSelect, Agilent), targeted against an in-house panel of 360 cancer-related genes (Yates *et al.* 2015). Libraries were sequenced on a MiSeq instrument (Illumina) to an average depth of 131× (range, 75×–254×) for the tumours and 165× (range, 50×-272×) for normal blood.

### Data processing and bioinformatics analysis

Raw sequence data were aligned to the human reference genome (Build-UCSC hg19) using BWA (Li & Durbin 2009). Somatic substitutions and insertions/deletions were detected using CaVEMan and Pindel, respectively (Raine *et al.* 2015, Jones *et al.* 2016). Somatic mutations were validated by manual inspection in Integrative Genomics Viewer and the COSMIC database. Mutations were restricted to those affecting protein-coding regions. In order to provide a complete overview of the mutations in the 360 genes in GEP-NEN, the data set was not restricted to driver mutations. Allele-specific copy number analysis and estimation of purity and ploidy were performed using FACETS (Shen & Seshan 2016). GISTIC 2.0 (Mermel *et al.* 2011) was used to identify frequent amplifications and deletions. Targetable molecular alterations were identified based on a predefined list and, in addition, by application of the OncoKB database (Supplementary Methods and Supplementary Table 1). A prediction model for the classification of tumours into the categories LC-NEC or NET G3 was built, based on mutational status of nine genes (*APC*, *ATRX*, *BRAF*, *DAXX*, *KRAS*, *MEN1*, *MYO5B*, *SMAD2* and *TP53*). Classification was performed using C5.0 decision tree algorithm implemented in R package C50 (v0.1.2; Supplementary files 1, 2 and 3) (https://www.rulequest.com/).

### Statistics

Statistical analyses were performed using R software (v3.5.1). Differences in mutation frequency between groups were assessed by odds ratio estimates with 95% CIs and by Fischer's exact test. Overall survival was assessed from the date of diagnosis to the date of death or last follow-up. Survival curves were drawn by the Kaplan–Meier method, and differences within groups were assessed by log-rank tests. *P* -values are given as two-sided, and *P* -values < 0.05 considered statistically significant.

## Results

From the Nordic GEP Registry biobank (see Supplementary Methods), samples with macroscopically sufficient tumour tissue to perform NGS (*n* = 279) were identified. Cases lacking normal tissue (*n* = 56), lacking slides for re-evaluation (*n* = 2) or reassessed as NET G2 (*n* = 1), adenocarcinoma (*n* = 14), mixed neuroendocrine neoplasms (MiNEN; *n*  = 23) or ambiguous neuroendocrine morphology concerning differentiation (*n* = 11) were excluded, resulting in a preliminary sample set of *n*  = 172. In the second, blinded, pathological review of 68 cases (59 out of the 172 cases with an initial NET/NEC separation and 9 out of the 11 uncertain), based on consensus between the three pathologists, four cases were reclassified (2 NEC cases were re-classified to NET G3 and 2 NET G3 were reclassified to NEC). In addition, five cases were re-classified from large cell to small cell, and two cases were re-classified from small cell to large cell. All nine cases with initial uncertain morphology were now re-classified as NET G3 or NEC. Thus, 181 patients were included for analyses. Among these were 152 neuroendocrine carcinomas (NEC) and 29 neuroendocrine tumours (NET G3), reflecting an expected distribution between NEC and NET G3.

A majority (67.4%) of samples were collected by biopsy, while the remaining specimens were collected by resection. Eighty per cent were stage IV, while 20% were stages I–III. The most frequent sites of primary tumour were the colon, rectum, esophagus, gastric and pancreas. Out of 25 cases with unknown primary, 22 had liver metastases and 5 of these had additional lung metastases, 8 bone metastases and no cases of brain metastasis. The three cases without liver metastases had only intra-abdominal lymph node metastases. Details of the basic patient characteristics are listed in Table 1.

**Table 1:** Patient characteristics.

Characteristic	Subgroup	Total NEN (*n* = 181, %)	NEC (*n* = 152, %)	NETG3 (*n* = 29, %)
Age				
	<50	13 (7)	9 (5.9)	4 (13.8)
	50–59	20 (11.6)	18 (11.8)	2 (6.9)
	60–69	64 (35.4)	55 (36.2)	9 (31)
	70+	84 (46.4)	70 (46.1)	14 (48.3)
Sex				
	Male	109 (60.2)	94 (61.8)	15 (51.7)
	Female	72 (39.8)	58 (38.2)	14 (48.3)
Site				
	Right colon	38 (21)	36 (23.7)	2 (6.9)
	Rectum	37 (20.4)	36 (23.7)	1 (3.4)
	Esophagus	19 (10.5)	18 (11.8)	1 (3.4)
	Gastric	17 (9.4)	16 (10.5)	1 (3.4)
	Unknown	25 (13.8)	19 (12.5)	6 (20.7)
	Pancreas	25 (13.8)	13 (8.6)	12 (41.4)
	Left colon	10 (5.5)	9 (5.9)	1 (3.4)
	Gallbladder/duct	3 (1.7)	3 (2)	0 (0.0)
	Other	3 (1.7)	2 (1.3)	1 (3.4)
	Small bowel	4 (2.2)	0 (0.0)	4 (13.8)
Cell type				
	Large cell		87 (57.2)	
	Small cell		65 (42.8)	
Ki-67				
	21–55%	36 (19.9)	13 (8.6)	23 (79.3)
	>55%	132 (72.9)	130 (85.5)	2 (6.9)
	>20% (exact value not specified)	13 (7.2)	9 (5.9)	4 (13.8)
Surgery of primary tumour				
	Resected (prior to sampling)	59 (32.6)	50 (32.9)	9 (31)
	Not resected	122 (67.4)	102 (67.1)	20 (69)
Disease				
	Non-metastatic (stage I–III)	36 (19.9)	31 (20.4)	5 (17.2)
	Metastatic (stage IV)	145 (80.1)	121 (79.6)	24 (82.8)
Smoking habit				
	Smoker	37 (20.4)	33 (21.7)	4 (13.8)
	Ex-smoker	54 (29.8)	45 (29.6)	9 (31)
	Non-smoker	73 (40.3)	60 (39.5)	13 (44.8)
	Unknown	17 (9.4)	14 (9.2)	3 (10.3)

### Molecular landscape of GEP-NEC

**Figure 1:**
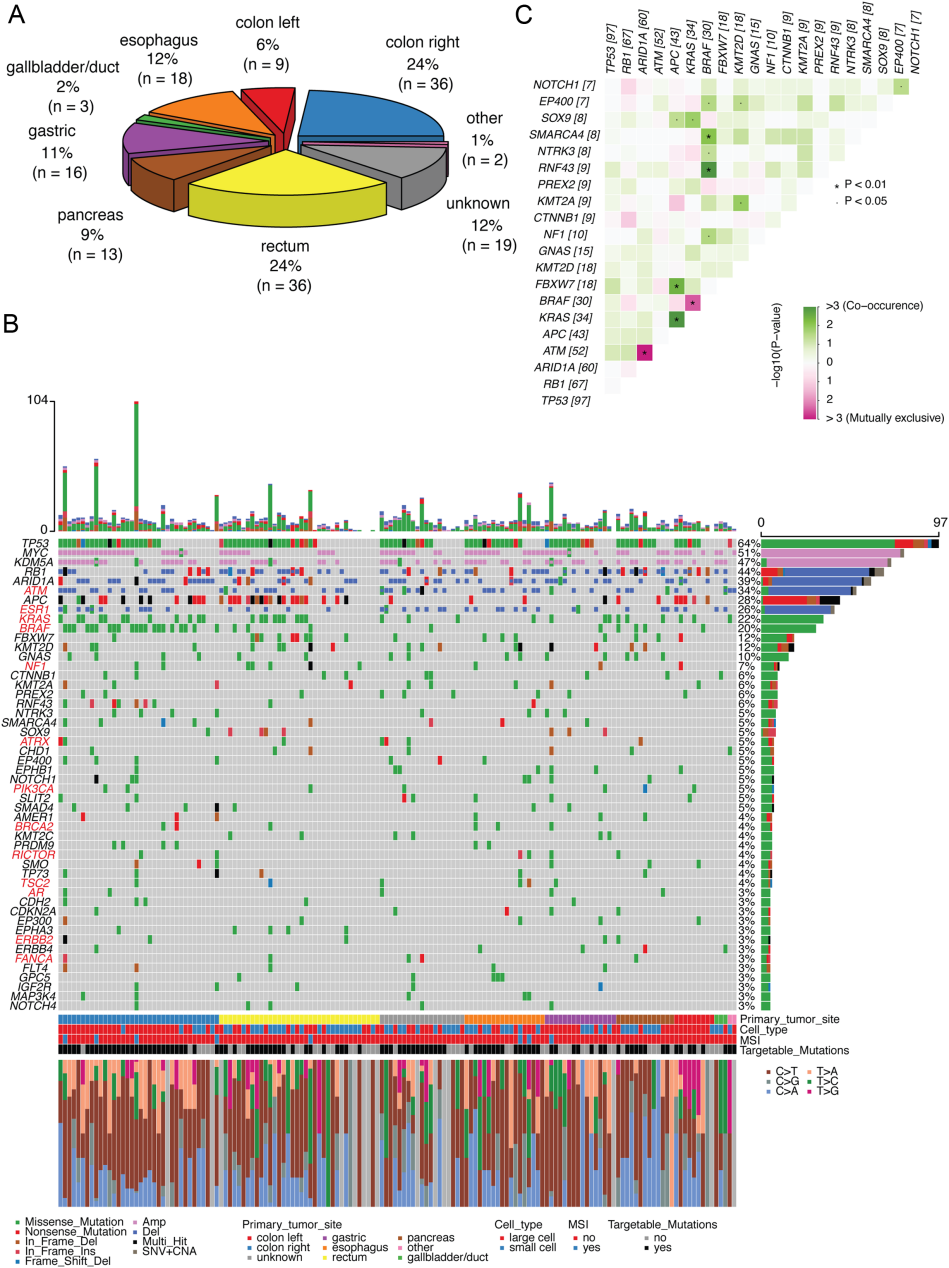
(A) Distribution of primary tumour site for the included cases of neuroendocrine carcinomas (NECs) cohort. (B) Oncoplot showing the top 50 most frequently altered genes (rows) among 152 NEC patients (columns). Upper panel shows the mutational burden per sample. Percentages on the right represent mutations frequency per gene. Genes with potentially targetable alterations are highlighted in red font (additional targetable mutations were observed among genes less frequently altered. The plot shows both potentially targetable and non-targetable alterations for these genes; for example, deletions of *ESR1* and non-V600E mutations of *BRAF* are not considered as targetable). The panel under the oncoplot area is composed of four single row heatmaps showing in order, from top to bottom, primary tumour site, cell type, MSI status and presence of one or more potentially targetable mutation. Stacked barplot (at the bottom) shows the fraction of nucleotide changes in each sample. 'Multi_hit' indicates that more than one mutation occurs in the same gene, in the same patient. (C) Co-occurrence and mutual exclusiveness of mutations in NEC patients. Co-occurring mutations are indicated by green squares and mutually exclusive mutations between gene pairs in purple. The color intensity is proportionate to the –log_10_ (*P* -value). *P -*value*s* were determined using Fisher’s exact test.

The 152 cases of GEP-NEC included different primary tumour sites (Fig. 1A). Assessing somatic point mutations and small insertions/deletions (indels), the most frequently mutated genes were *TP53* (64%), *APC* (28%), *KRAS* (22%), *BRAF* (20%) and *RB1* (14%) (Fig. 1B). We found a rather narrow range of other genes (*KMT2D*, *FBXW7*, *GNAS*, *ARID1A*, *NF1* and *CTNNB1*) harbouring mutations in 6–12% of the patients and a long tail of cancer genes with mutations in <6%.

**Figure 2:**
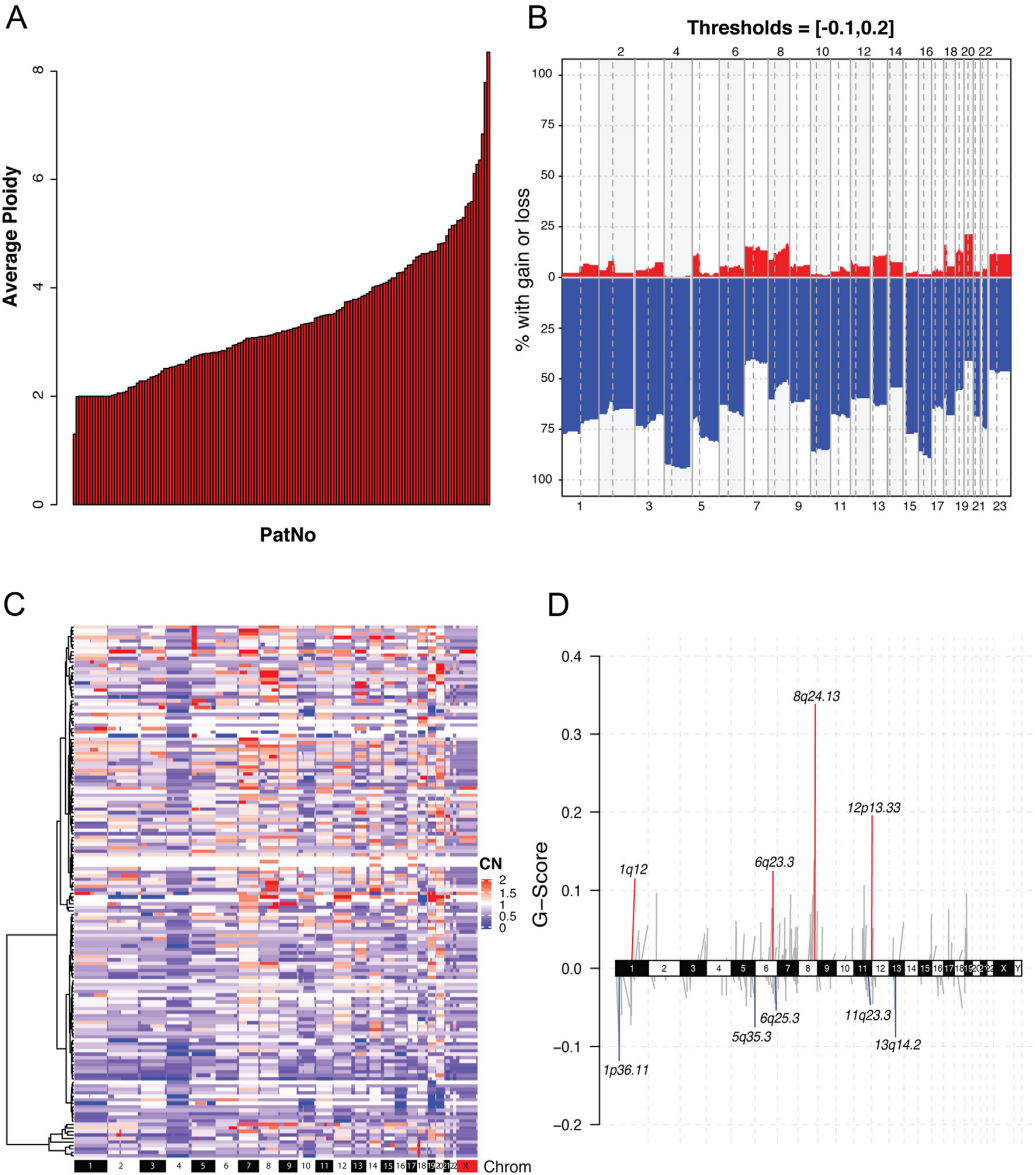
(A) Stacked barplot illustrating the average ploidy in each of the NEC samples. (B) Frequency of copy number aberrations in the NEC cohort (*n* = 152). Y-axis indicates the fraction of patients with copy number losses (blue) and gains (red) across the genome. Chromosome numbers are indicated on the x-axis. Chromosomes and chromosome arms are separated by vertical lines. (C) Heatmap representing the copy number alterations for each segment, relative to average genome ploidy for each sample. Unsupervised hierarchical clustering of patients on the y-axis and chromosomes on the x-axis. (D) Significant copy-number gains (red) and losses (blue) identified by two-sided hypothesis testing using GISTIC2.0, corrected for multiple hypothesis testing. Significant regions (chromosome locus and focal copy number changes) for known cancer-associated genes are labeled.

The average ploidy in the analysed NECs was 3.44, indicating that a large fraction had undergone whole-genome duplication (WGD; Fig. 2A). The vast majority of copy number alterations (CNA) were deletions/copy number losses, presumably events occurring after WGD (Fig. 2B, C and D). The most frequently deleted chromosomal regions were 1p36.11 containing *ARID1A* (35%), 6q25.3 containing *ESR1* (25%), 11q23.3 containing *ATM* (31%) and 13q14.2 containing *RB1* (34%). The most frequently gained/amplified regions were 8q24.13 containing *MYC* (51%) and 12p13.33 containing *KDM5A* (45%) (Fig. 1B).

We found a high co-occurrence of mutations in *APC* and* KRAS* (*P* = 4.3 × 10^−8^; Fig. 1C). *APC* mutations also co-occurred with *FBXW7* mutations (*P* = 3.6 × 10^−3^). *BRAF* mutations co-occurred with both mutations in *RNF43* and *SMARCA4* (*P* = 1.7 × 10^−4^ and 8 × 10^−3^), while being mutually exclusive to *KRAS* mutations (*P* = 3.2 × 10^−3^). *ATM* deletions were mutually exclusive to *ARID1A* deletions (*P* = 8.9 × 10^−4^). Several known pathways were affected by alterations in >20% of the cohort, including DNA damage response, Wnt/beta-catenin, RAS GTPase, ERK signalling, receptor tyrosine kinase upstream of RAS and Notch signalling (Supplementary Fig. 1). Regarding proliferation phenotype, despite the small number of NEC with Ki-67 21–55% (*n* = 13) compared to NEC with Ki-67 > 55% (*n* = 130), CNA in *MYC* was the only significant difference (enrichment) seen in NEC with Ki-67 21–55% (*P* = 0.018; Supplementary Fig. 2). CNA in *ARID1A* and mutations in *FBXW7* were significantly enriched among non-smokers as compared to smokers (*P* < 0.05; Supplementary Fig. 3).

On average, the number of mutations within the targeted gene panel was 7.7 (range, 0–104), corresponding to a tumour mutation burden (TMB) of 5.1 per MB (range, 0–69; Supplementary Table 2).

Importantly, we assessed the fraction of NEC samples harbouring molecular alterations that could potentially be targetable, based on a predefined list of SNVs and CNAs (see Methods; Supplementary Table 1). Among the 152 samples, we found 101 (66%) with one or more alterations that could be targeted by available drugs. The majority of these alterations were related to defects in DNA repair, making tumours potentially sensitive to PARP inhibition, but frequent targetable alterations were also seen in *BRAF*, MTOR signalling as well as several other genes (Fig. 1 and Supplementary Table 1). In addition to the analysis of potentially targetable mutations, we performed a highly stringent assessment of those alterations listed as established biomarkers in the OncoKB database. Even in this restricted analysis, as many as 22% of the tumours harboured one or more targetable alterations.

### Specific molecular features of GEP-NEC according to primary tumour site

**Figure 3:**
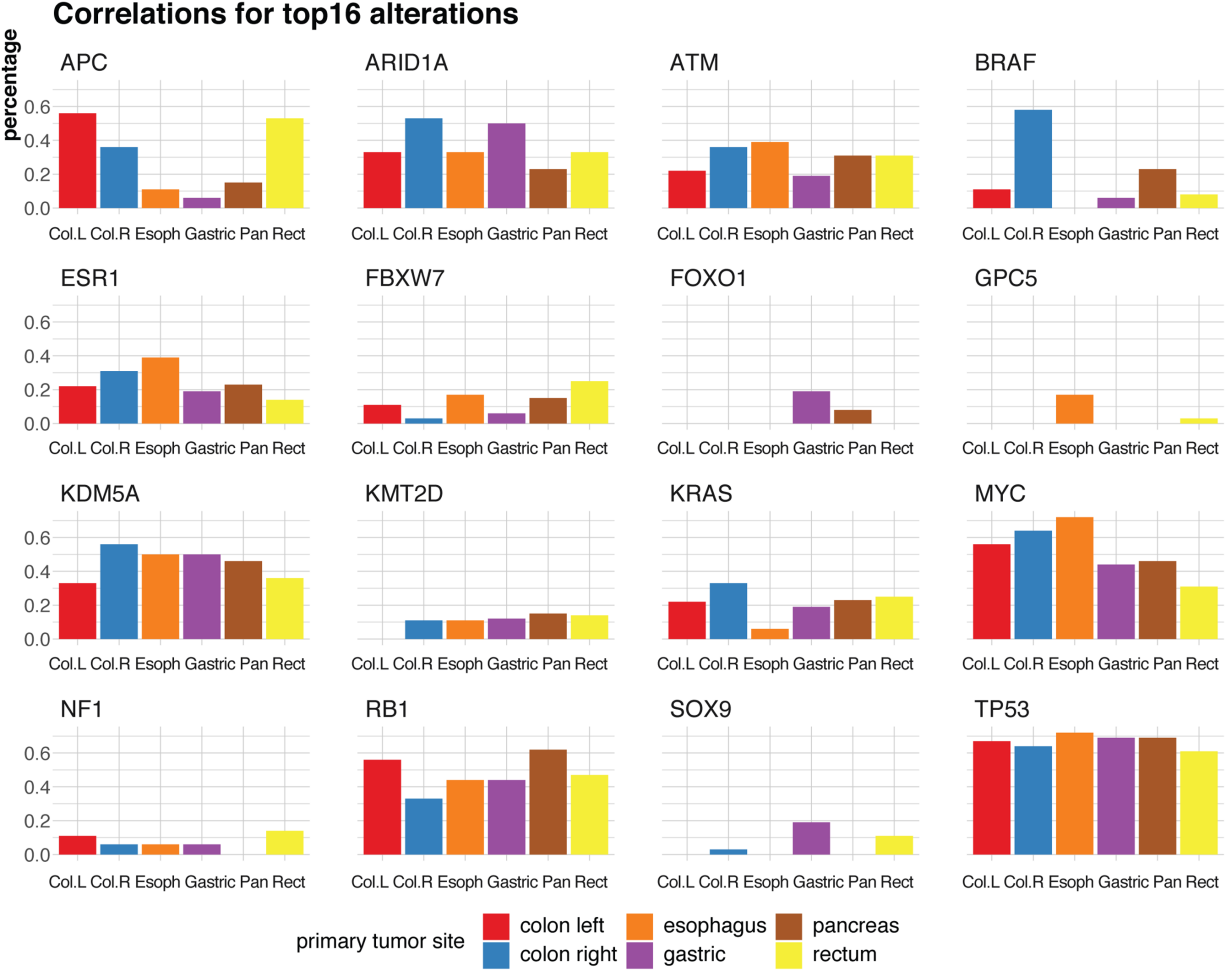
Barplots indicating mutation frequency for the top 16 frequently altered genes in NEC patients, stratified for the six primary tumour sites (left colon, right colon, esophagus, gastric, pancreas, rectum). Y-axis shows the frequency (in percentage) of the alteration for each site.

The frequency of genetic alterations varied according to primary NEC tumour site (Fig. 3). In colonic primaries (*n* = 45), mutations were frequent in *TP53* (64%), *BRAF* (49%), *APC* (40%) and *KRAS* (31%) (Fig. 3 and Supplementary Fig. 4A). Regarding CNA, we found loss of *ARID1A* (42%), *RB1* (24%), *ATM* (33%) and *ESR1* (27%), while amplification was found for *KDM5A* (49%) and *MYC* (62%). *BRAF* was mutually exclusive to *KRAS* and *APC* mutations (*P* = 6.8 × 10^−6^ and 4.6 × 10^−6^, respectively), while the two latter were significantly co-occurring (*P* = 1.4 × 10^−6^; Supplementary Fig. 4B).

In rectal primaries (*n* = 36), we found mutations in *TP53* (61%), *APC* (53%), *FBXW7* (25%) and *KRAS* (25%) (Fig. 3 and Supplementary Fig. 5A). Gene alterations in the range of 8–14% were limited to *KMT2D*, *NF1, EPHA3*, *SOX9* and *BRAF*. Regarding CNA, we found loss of *RB1* (39%), *ARID1A* (25%), *ATM* (28%) and *ESR1* (14%), while amplifications were seen for *KDM5A* (33%) and *MYC* (31%). Mutations of *APC* and *KRAS* both co-occurred with *TP53* mutations (*P* = 5 × 10^−3^ and *P* = 6 × 10^−3^; Supplementary Fig. 5B). Thus, the frequency of alterations in different genes was fairly similar comparing colonic and rectal origin, with the exception of *BRAF* mutations, which were significantly more frequent in colonic NEC (*P* = 8.0 × 10^−5^; Fig. 3).

Among the remaining tumours, the most frequently altered genes in gastric NEC (*n* = 16) were *TP53* (69%), *ARID1A* (50%), *RB1* (44%) and *KDM5A* (50%). Among genes that were not frequently mutated in other primary sites, three patients had *FOXO1* mutations (19%) and three *SOX9* mutations (19%). In oesophageal NEC (*n* = 18) *TP53* was mutated in 72%, and *MYC* amplified in 72%. Nine of 13 pancreatic NEC had *TP53* mutations (69%), 8 had *RB1* mutations and/or deletions (62%). *BRAF* and *KRAS* were each mutated in three patients (23%; Supplementary Figs 6, 7 and 8). Thus, we found some primary tumour site-specific molecular differences. *BRAF* mutations were most common in colorectal primaries compared to other sites (*P* = 2.9 × 10^−8^).

### Large cell and small cell GEP-NEC

**Figure 4:**
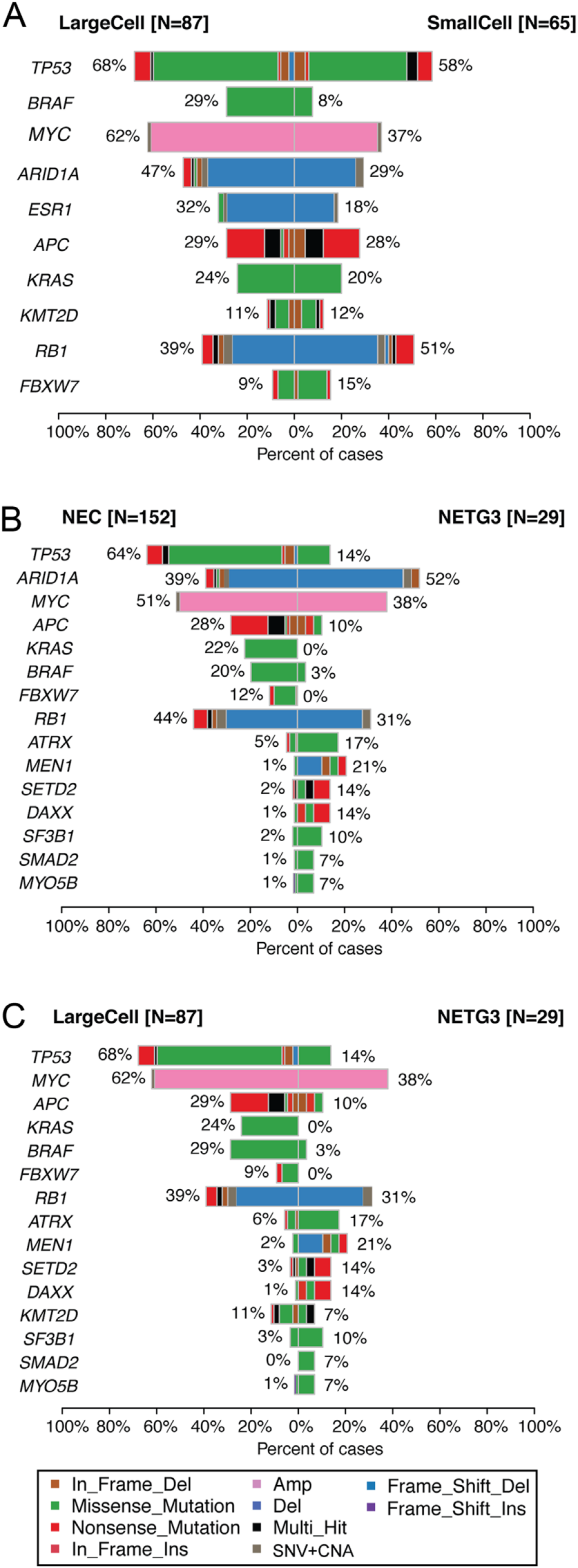
Co-bar plots illustrating the differences in mutation frequencies for the most frequently mutated genes, between (A) large cell NEC (*n* = 87) vs small cell NEC (*n* = 65), (B) NEC (*n* = 152) vs NET G3 (*n* = 29) and (C) large cell NEC (*n* = 87) vs NET G3 (*n* = 29).

Comparing large cell (LC)-NEC to small cell (SC)-NEC, we found alterations in *BRAF*, *MYC* and *ARID1A* to be significantly enriched among LC-NEC (Fig. 4A and Supplementary Figs 9, 10, 11). Mutations in the *MAP3K1* gene were somewhat enriched in SC-NEC.

### GEP NET G3

Among 29 NET G3, we found *ARID1A* copy number loss in 14 cases (48%) (Supplementary Fig. 12). Other frequently altered genes were *ATM* (*n* = 14; 48%), *ESR1* (*n* = 11; 38%), *KDM5A* (15; 52%), *MYC* (11; 38%), *RB1* (9; 31%), *MEN1* (6; 21%) and *ATRX* (5; 17%). In the subset of pancreatic NET G3 (*n* = 12), we found mutations in *MEN1* (*n* = 4), *TP53* (*n* = 3), *DAXX* (*n* = 3) and *ATRX* (*n* = 3; Supplementary Fig. 12).

On average, the number of mutations in NET G3 was 6.9 (range, 0–89), corresponding to a tumour mutation burden (TMB) of 4.6 per MB (range, 0–59; Supplementary Table 2).

Out of the 29 NET G3 samples, we found 21 (72%) to harbour one or more potentially targetable alterations (Supplementary Table 1).

### Molecular differences between NEC and NET G3

We observed a numerical, but nonsignificant, difference in the number of genetic alterations among NEC as compared to NET G3 (average: 9.9 vs 9.4, *P* > 0.05; Supplementary Table 2). However, we found alterations in several genes to be significantly skewed between the two groups (Fig. 4B and Supplementary Fig. 13). Mutations in *TP53* and *KRAS* were significantly enriched among NEC (*P* = 7.0 × 10^−7^ and 0.003). Mutations in *APC, BRAF* and *FBXW7* were also enriched in NEC, although not statistically significant. Alterations in *MEN1*, *DAXX*, *SETD2* and *ATRX* were significantly enriched in NET G3. Given that SC carcinoma per definition is NEC, the subgroups important to distinguish are LC-NEC from NET G3. We therefore restricted our analyses to genes differentially altered between the two latter groups. Alterations in *TP53*, *BRAF*, *MYC* and* KRAS* were significantly enriched among LC-NEC (Fig. 4C and Supplementary Fig. 14). *MEN1* and* DAXX* mutations were significantly enriched in NET G3.

Testing whether molecular alterations of LC-NEC could be a classifier to distinguish them from NET G3, we found 72/87 LC-NEC having mutations in *APC*, *TP53*, *KRAS* or *BRAF* as compared to 6/29 NET G3 (*P* = 2.3 × 10^−9^). This crude four-gene classifier yields 92.3% sensitivity, 60.5% specificity and a positive predictive value of 82.8% for distinguishing LC-NEC from NET G3. We also applied a more refined approach, including genes enriched for mutations either in LC-NEC or NET G3 and built a prediction model based on decision tree classification (https://www.rulequest.com/). Although the model must be interpreted with caution since no validation sample set was available, applying the mutation distribution of the nine genes (*APC*, *TP53*, *KRAS*, *BRAF, ATRX, DAXX, MEN1, MYOB5B* and*SMAD2*), we generated a classifier yielding 86.8% sensitivity, 88.9% specificity and a positive predictive value of 96.0% (Supplementary files 1, 2 and 3).

### Microsatellite instability (MSI) in GEP-NEC

MSI was seen in 8/152 GEP-NEC (5.3%) and in only 1 of 29 NET G3 (3.4%). We found a slight MSI enrichment in colonic GEP-NEC: four colonic primaries displayed MSI (8.9% of colon cases) while two were oesophageal, one rectal and one gastric (Fig. 1B). In line with previous findings in colorectal adenocarcinoma (Aasebo *et al.* 2019), we found MSI to co-occur with *BRAF* mutations in colorectal NEC (4/4 MSI harboured *BRAF* mutation). Also, as expected, the eight tumours with MSI were the ones with the highest number of mutations among the NEC cases (Fig. 1B).

### Prognosis

Overall survival (OS) for GEP-NEC patients was significantly worse than for NET G3 patients (11 vs 18 months; *P* = 0.049; Supplementary Fig. 15). Within the NEC group, patients with SC-NEC had a significantly worse OS than those with LC-NEC (9 vs 12 months; *P* = 0.025; Supplementary Fig. 15). Although trends for the prognostic value of key features such as MSI and TMB were observed, these did not reach statistical significance in the present data set. Neither did mutation status for any of the investigated single genes (in univariate analyses).

## Discussion

Based on morphological similarities with small-cell lung cancer (SCLC), SCLC chemotherapy schedules are used both in the adjuvant and in metastatic setting for GEP-NEC. Thus, platinum/etoposide chemotherapy is the cornerstone in adjuvant and palliative treatment of GEP-NEC (Strosberg *et al.* 2010, Garcia-Carbonero *et al.* 2016, Pavel *et al.* 2020), although the benefit of adjuvant therapy for GEP-NEC has never been proven and as many as 1/3 of metastatic patients have immediate disease progression on such treatment, and in general, PFS and OS are short (Sorbye *et al.* 2013, Garcia-Carbonero *et al.* 2016, Sorbye *et al.* 2019). A confirmation of similar molecular patterns in GEP-NEC and SCLC would support the current treatment extrapolation. However, our data show that this is not the case. In SCLC, *TP53* mutations are reported in 86–93% and *RB1* mutations in 40–62% (Peifer *et al.* 2012, Dowlati *et al.* 2016, Miyoshi *et al.* 2017, Eskander *et al.* 2020) while biallelic inactivation of *TP53* and *RB1* (by any mechanism) is a universal finding for all SCLC (George *et al.* 2015). The 2019 WHO classification for digestive tumours state that GEP-NEC frequently have *TP53* and *RB1* mutations (WHO 2019). Until recently, only small series have reported *TP53* and *RB1* alterations in GEP-NEC, and reports are methodologically inconsistent as some studies report gene mutations, others genetic alterations in general and some altered protein levels by immunohistochemistry. *TP53* mutations in GEP-NEC are found in 57–59% (Vijayvergia *et al.* 2016, Busico *et al.* 2020), in colorectal NEC in 50–84% (Shamir *et al.* 2019, Capdevila *et al.* 2020) and in 8/12 pancreatic NEC (Konukiewitz *et al.* 2018). We found *TP53* mutations in 64% of GEP-NEC, 68% in large-cell and 58% in small-cell, which are substantially fewer than observed in SCLC. We found *RB1* mutations in only 14% of cases, strikingly less than observed in both SCLC and LCLC. However, we did find a substantial fraction of cases with copy number loss of the *RB1* locus, and as such, *RB1* alterations were observed in 34% of our cases. These results could indicate a lower mutation frequency of both *TP53* and *RB1* in GEP-NEC than in SCLC/LCLC. It is important to note that some previous studies finding high frequencies of *RB1* inactivation have based their 'mutation calling' on lacking pRb staining by IHC. Copy number losses or epigenetic silencing, in addition to mutations, are likely to have caused such a lack of protein expression in many cases. Overall, our results clearly indicate that *RB1* mutations are relatively rare in GEP-NEC, whereas copy number losses are more frequent. We argue that this should be specified in future efforts to establish classification guidelines. Comparing our findings to LCLC, another major difference was *STK11* and *KEAP1* alterations. In LCLC, 33% *STK11* and 29% *KEAP1* mutations are observed (Rekhtman *et al.* 2016), whereas we found *STK11* mutations in only two LC-NEC (2.3%) and *KEAP1* mutations in three (3.4%). *KRAS* mutations have been reported in 4–22% of LCLC (Rekhtman *et al.* 2016, Miyoshi *et al.* 2017), whereas *BRAF* mutations are rare; 0–3% in LCLC (Miyoshi *et al.* 2017, George *et al.* 2018) and 1/110 SCLC-cases (George *et al.* 2015). We found *KRAS* and *BRAF* mutations in 22% and 20% of GEP-NEC, respectively.

Although we estimated tumour mutation burden from a targeted panel of 360 genes, such estimates have been shown to strongly reflect the burden detected in larger-scale sequencing (Chalmers *et al.* 2017). The limited mutation burden (median 5.1 per MB) we observed in GEP-NEC is in contrast to pulmonary NEC and may explain the limited benefit of checkpoint inhibitors.

Taken together, based on these molecular differences, we believe that one must be careful when basing treatment decisions for GEP-NEC on results from pulmonary NEC (SCLC/LCLC). We argue that other treatment strategies should be exploited in GEP-NEC. Strategies using adenocarcinoma schedules may be an option, but most importantly, our results show that as many as 66% of GEP-NEC cases have potentially targetable molecular alterations. This is above the pan-cancer average (57%), reported by Bailey *et al.* (2018). Although the analyses are not directly comparable, our data at least indicate that the fraction of targetable mutations is higher in GEP-NEC than most cancers. Considering the very limited results of first and second line chemotherapy for GEP-NEC (Sorbye *et al.* 2013, 2019, Garcia-Carbonero *et al.* 2016), the possibility to apply targeted therapy in GEP-NEC thus seems to be a major possibility to improve the very poor prognosis of these patients. Extrapolation from SCLC has also been a common practice for the treatment of all extrapulmonary neuroendocrine carcinomas, but a recent report shows major genetic differences between SCLC and neuroendocrine cervical carcinoma (HGNECC), further questioning such a general extrapolation (Eskander *et al.* 2020). The HGNECC genetics are also different compared to our GEP-NEC findings, illustrating that extrapulmonary NEC should not be assessed as a joint group but rather according to primary tumour site.

In contrast to the large molecular differences reported between SCLC and LCLC, we only found some differences between small cell and large cell GEP-NEC; especially more frequent *BRAF* mutations in colonic large-cell NEC were observed. How these results could affect the clinical practice of treating small-cell and large-cell GEP-NEC similarly (Strosberg *et al.* 2010, Garcia-Carbonero *et al.* 2016) needs to be studied further. In a previous study (Busico *et al.* 2020), molecular differences within NEC were found according to the proliferation rate. In our present data, we only found minor differences between tumour with high vs low Ki-67. In contrast to the many genetic differences seen between smokers and nonsmokers in lung cancer (Chapman *et al.* 2016, Smolle & Pichler 2019), only minor differences were found between these groups in our study illustrating that GEP-NEC is a specific entity.

In our study, molecular alterations varied in some aspects between the different primary tumour sites. *TP53* mutations were quite similarly distributed in all primary sites. *RB1* alterations were especially seen in pancreatic (62%) and left colonic (56%), and less in right colonic NEC. Prior studies have shown the loss of pRb protein expression in 67–80% of GEP-NEC (Li *et al.* 2008, Busico *et al.* 2020), 33–55% of pancreatic NEC (Hijioka *et al.* 2017, Konukiewitz *et al.* 2018) and 56% of colorectal (CR)-NEC (Takizawa *et al.* 2015). In our study, right-sided colon NEC was the only primary tumour site with a high number of *BRAF* V600E (70%) mutations. *BRAF* V600E mutations were reported in prior colorectal NEC/MINEN series in a frequency of 28–47% (Dizdar *et al.* 2019, Capdevila *et al.* 2020). The combination of a BRAF and EGFR inhibitor was recently approved for metastatic colorectal adenocarcinoma (Van Cutsem *et al.* 2019), and case reports have shown the benefit of such treatment in CR-NEC (Klempner *et al.* 2016). Our results also illustrate the possible importance of sidedness in CR-NEC, as shown for CR adenocarcinoma (Tejpar *et al.* 2017). We found *KRAS* mutations at all sites at an incidence of 19–33%, less among oesophageal primaries. This is partly in line with previous studies revealing *KRAS* mutations in gastric (6%), pancreatic (49%) and colonic (48%) NEC/MINEN (Sahnane *et al.* 2015, Hijioka *et al.* 2017, Capdevila *et al.* 2020). A recent study reported that EGFR blockade reverts resistance to *KRAS* G12C inhibition in colorectal adenocarcinoma (Amodio *et al.* 2020). However, we only found five *KRAS* G12C mutations in our study.

Recently, a large study compared genetic differences between gastrointestinal and pancreatic NEN and between low-grade and high-grade GEP-NEN (Puccini *et al.* 2020). One hundred and thirty-five HG tumours showed mutations in *TP53* (51%), *KRAS* (30%), *RB1* (11%) and *BRAF* (5%). The authors concluded that *RB1* and *TP53* mutations are frequent in HG GEP-NEN. Compared to NET this is correct, however, their results confirm our result that *RB1* mutations are much rarer in GEP-NEC compared to SCLC. In contrast to our study, this study neither included pathological re-evaluation regarding the separation of NET G3 from NEC nor filtered for germline variants. The low *BRAF* mutation frequency compared to our present data may be due to fewer colon NECs; however, primary sites were not specified. In our study, we found MSI in eight NEC cases (5.3%). In prior studies, the frequency of MSI in GEP-NEC/MINEN is reported to be 0–15% (La Rosa *et al.* 2012, Sahnane *et al.* 2015, Chapman *et al.* 2016, Puccini *et al.* 2020). MSI seems to be an agnostic tumour marker for the benefit of checkpoint inhibitors (Petrelli *et al.* 2020), and at least CR-NEC should be tested for MSI with the potential to guide treatment choice.

In our HG GEP-NEN cohort, we found a NET G3 incidence of 16%, in accordance with the 10–18% observed in prior studies (Heetfeld *et al.* 2015, Sorbye *et al.* 2019, Elvebakken *et al.* 2021). In NET G3, which is most frequently occurring in the pancreas (Coriat *et al.* 2016), most alterations were also seen in pancreatic primaries, including mutations in *MEN1* (33%), *ATRX* (25%) and *DAXX* (25%; within the subgroup of pancreatic NET G3). This is roughly in line with a previous study of 20 pancreatic NET G3 where 3 *ATRX* and 7 *DAXX* mutations were found (Tang *et al.* 2016*a*). Although the number of cases is low, these results seem similar to pancreatic NET G1-G2 where relative frequencies of genetic alterations were reported as follows: *MEN1* (24–44%), *DAXX* (11–25%) and (*ATRX* 16–33%) (Jiao *et al.* 2011, Chan *et al.* 2018, Puccini *et al.* 2020). We found fewer alterations in NET G3 compared to NEC. *TP53* was mutated in 14% of the NET G3 cases and 31% had *RB1* alterations, while no *KRAS* mutations were found. Notably, only one NET G3 sample was found to be MSI-positive. There are few other studies on molecular alterations in NET G3. In a study on 15 GEP-NET G3, absent pRB staining was seen in 9/15 cases (60%), whereas only 3 genomic alterations were found (in *ATM*, *VHL* and *IDH1*) (Busico *et al.* 2020). In a study of 21 pancreatic NET G3, neither abnormal pRb expression nor *KRAS* mutations were found (Hijioka *et al.* 2017). A third study reported 1 single *TP53* mutation among 11 pancreatic NET G3 (Konukiewitz *et al.* 2018). These results strongly support that GEP-NEC and NET G3 are different diseases and that the clear separation of these entities is important. Treatment used for GEP-NEC should probably not be used for NET G3 patients without careful consideration. The molecular differences could in part explain why NET G3 patients have less response to platinum-based chemotherapy than NEC (Sorbye *et al.* 2019).

Separation of NET G3 from NEC based on morphology can be challenging. Some have suggested to use *MEN1*/*ATRX*/*DAXX* and *RB1*/*TP53* to aid in separation of these entities (Tang *et al.* 2016*a*). The ESMO 2020 GEP-NEN guidelines suggest using *RB1* mutations or *RB1* loss to discriminate between NET G3 and NEC (Pavel *et al.* 2020). Our data highlight that testing for *RB1* loss would perform better than *RB1* mutations in such an approach. Applying molecular data, we assessed the main differences between NEC and NET G3. Given that SC tumours per definition are NEC, we considered the subgroups that are clinically important to distinguish to be LC-NEC from NET G3. Mutations in *APC*, *TP53*, *KRAS* and *BRAF* were enriched in LC-NEC as compared to NET G3, and the mutation status of these genes yielded a classifier with surprisingly high sensitivity, specificity and positive predictive value. Applying a more refined prediction model including nine genes, we found even better prediction scores. Notably, these models must be interpreted with caution, since we currently do not have an independent data set for validation analyses. However, these calculations indicate a possibility to apply molecular data for the classification of cases.

An important strength of our study is that scans (haematoxylin and eosin, synaptophysin, chromogranin-A and Ki-67-stain) of all NEN were centrally reevaluated histologically according to the most recent 2019 WHO classification. Furthermore, for all patients, we had available DNA from the blood, enabling proper filtering of genetic variants to identify true somatic alterations. In contrast to many prior studies, we excluded MiNEN, avoiding possible inclusion of the adenocarcinoma part in the DNA extraction. We did not include other extrapulmonary NEC and as such, ensured a relatively homogeneous sample of GEP-NEC.

Regarding limitations, our cohort includes the largest number of cases reported to date, but a large fraction of cases were colon (24%) and rectal (24%) primaries, resulting in other subgroups with limited representation. Thus, it may be that even larger sets of samples are needed to get the full overview of the mutational landscape of each individual subgroup. Further, it is important to note that the sample set may have a bias in terms of the patient population. About one-third of patients in the present sample set had undergone primary tumour surgery, a much debated, but possible prognostic factor (Sorbye *et al.* 2015). Further, although our panel of 360 genes cover the most relevant cancer genes, it is clear that a more comprehensive overview of the mutational landscape, including structural rearrangement etc., would have been obtained by whole-genome sequencing and such detailed analyses are warranted for future.

In summary, we performed a comprehensive assessment of the molecular tumour alterations in a large series of gastroenteropancreatic high-grade neuroendocrine neoplasms. We found a marked difference in the molecular profile compared to prior results in SCLC and LCLC, some differences comparing large-cell and small-cell GEP-NEC, a profile variation according to primary tumour site, a high fraction GEP-NEC with targetable mutations pointing to novel important therapeutic strategies and a possible molecular strategy to separate NEC from NET G3.

## Supplementary Material

Supplementary Methods

Supplementary Table S1

Supplementary Table S2

Supplementary Table S3

Supplementary Table S4

Supplementary Table S5

Suppl. Figure 1. Pathway analysis of the NEC cohort. Coloured squares indicate genetic alterations, i.e single nucleotide variants (SNVs; red) and insertions/deletions (InDels;blue). Columns represent patients and rows represent pathways. Percentage referring to the fractions of patients affected by somatic mutations involved in a given pathway [Suppl. Table 2]. Bars on the top indicate the number of pathways affected in a given patient, and type of variant InDel or SNV.

Suppl. Figure 2. Forest plot showing the enrichments for altered genes in patients with Ki67 21-55% relative to those with Ki67>55% (illustrated as odds ratio [OR] where OR<1 indicates enrichment in patients with Ki67 21-55% and OR>1 indicates enrichment in patients with Ki67>55%). The plot incudes all genes mutated in minimum of 3 of the patients.

Suppl Figure 3. Forest plot showing the enrichments for altered genes in smokers versus non-smokers among NEC patients (illustrated as odds ratio [OR] where OR<1 indicates enrichment in smokers and OR>1 indicates enrichment in non-smokers). The plot incudes all genes mutated in minimum of 3 of the patients.

Suppl Figure 4. A) Oncoplot showing the top 50 most frequently altered genes (rows) among 45 colonic NEC primaries (columns). Upper panel shows the mutational burden per sample. Percentages on the right represent mutations frequency per gene. The panel under the oncoplot area is composed of 3 single row heatmaps showing in order, from top to bottom, primary tumor site, cell type and MSI status. “Multi_hit” indicates that more than one mutation occurs in the same gene, in the same patient. B) Co-occurrence and mutual exclusiveness of mutations in colonic NEC primaries. Co-occurring mutations are indicated by green squares and mutually exclusive mutations between gene pairs in purple. The color intensity is proportionate the –log10 (p-value). p-values were determined using Fisher’s exact test.

Suppl Figure 5. A) Oncoplot showing the top 50 most frequently altered genes (rows) among 36 rectal NEC primaries (columns). Upper panel shows the mutational burden per sample. Percentages on the right represent mutations frequency per gene. The panel under the oncoplot area is composed of 3 single row heatmaps showing in order, from top to bottom, primary tumor site, cell type and MSI status. “Multi_hit” indicates that more than one mutation occurs in the same gene, in the same patient. B) Co-occurrence and mutual exclusiveness of mutations in rectal NEC primaries. Co-occurring mutations are indicated by green squares and mutually exclusive mutations between gene pairs in purple. The color intensity is proportionate the –log10 (p-value). p-values were determined using Fisher’s exact test.

Suppl Figure 6. Oncoplot showing the top 50 most frequently altered genes (rows) among 16 gastric NEC primaries (columns). Upper panel shows the mutational burden per sample. Percentages on the right represent mutations frequency per gene. The panel under the oncoplot area is composed of 3 single row heatmaps showing in order, from top to bottom, primary tumor site, cell type and MSI status. “Multi_hit” indicates that more than one mutation occurs in the same gene, in the same patient.

Suppl Figure 7. Oncoplot showing the top 50 most frequently altered genes (rows) among 13 pancreatic NEC primaries (columns). Upper panel shows the mutational burden per sample. Percentages on the right represent mutations frequency per gene. The panel under the oncoplot area is composed of 3 single row heatmaps showing in order, from top to bottom, primary tumor site, cell type and MSI status. “Multi_hit” indicates that more than one mutation occurs in the same gene, in the same patient.

Suppl Figure 8. Oncoplot showing the top 50 most frequently altered genes (rows) among 18 esophageal NEC primaries (columns). Upper panel shows the mutational burden per sample. Percentages on the right represent mutations frequency per gene. The panel under the oncoplot area is composed of 3 single row heatmaps showing in order, from top to bottom, primary tumor site, cell type and MSI status. “Multi_hit” indicates that more than one mutation occurs in the same gene, in the same patient.

Suppl Figure 9. A) Oncoplot showing the top 50 most frequently altered genes (rows) among 87 large cell NEC patients (columns). Upper panel shows the mutational burden per sample. Percentages on the right represent mutations frequency per gene. The panel under the oncoplot area is composed of 3 single row heatmaps showing in order, from top to bottom, primary tumor site, cell type and MSI status. “Multi_hit” indicates that more than one mutation occurs in the same gene, in the same patient. B) Co-occurrence and mutual exclusiveness of mutations in large cell NEC patients. Co-occuring mutations are indicated by green squares and mutually exclusive mutations between gene pairs in purple. The color intensity is proportionate the –log10 (p-value). p-values were determined using Fisher’s exact test.

Suppl Figure 10. A) Oncoplot showing the top 50 most frequently altered genes (rows) among 65 small cell NEC patients (columns). Upper panel shows the mutational burden per sample. Percentages on the right represent mutations frequency per gene. The panel under the oncoplot area is composed of 3 single row heatmaps showing in order, from top to bottom, primary tumor site, cell type and MSI status. “Multi_hit” indicates that more than one mutation occurs in the same gene, in the same patient. B) Co-occurrence and mutual exclusiveness of mutations in small cell NEC patients. Co-occuring mutations are indicated by green squares and mutually exclusive mutations between gene pairs in purple. The color intensity is proportionate the –log10 (p-value). p-values were determined using Fisher’s exact test.

Suppl. Figure 11. Forest plot showing the enrichments for altered genes in patients with the large cell relative to the small cell NEC (illustrated as odds ratio [OR] where OR<1 indicates enrichment in large cell NEC and OR>1 indicates enrichment in small cell NEC). The plot incudes all genes mutated in minimum of 3 of the patients.

Suppl.Figure 12. Oncoplot showing the top 50 most frequently altered genes (rows) among 29 NET–G3 patients (columns). Upper panel shows the mutational burden per sample. Percentages on the right represent mutations frequency per gene. The panel under the oncoplot area is composed of one single row heatmap showing in order primary tumor site. “Multi_hit” indicates that more than one mutation occurs in the same gene, in the same patient.

Suppl. Figure 13. Forest plot showing the enrichments for altered genes patients with NEC versus NET G3 (illustrated as odds ratio [OR] where OR<1 indicates enrichment in NEC and OR>1 indicates enrichment in NET G3). The plot incudes all genes mutated in minimum of 3 of the patients.

Suppl. Figure 14. Forest plot showing the enrichments for altered in patients with large cell NEC versus NET G3 (illustrated as odds ratio [OR] where OR<1 indicates enrichment in large cell NEC and OR>1 indicates enrichment in NET G3). The plot incudes all genes mutated in minimum of 3 of the patients.

Suppl. Figure 15. Kaplan-Meier overall survival analyses of A) NEC patients with MSI versus MSS, B) NEC according to mutational burden (i.e. number of mutations and alterations within the analyzed 360 cancer genes), below vs. above median (<8 vs. >=8), C) NEC versus NETG3, D) Large cell versus Small cell. P-values given are based on long-rank tests.

## Declaration of interest

H E has received honoraria from Bayer. G O H has received grants or honoraria from Ipsen Amgen, BMS, MSD, Roche and Bayer. S K has received grants or honoraria from Astra-Zeneca, Pfizer, Pierre-Fabre, Novartis, Sobi, Amgen, Sanofi Aventis and Roche. H S has received research support from Novartis, Amgen, Ipsen and honoraria from Novartis, Ipsen, Pfizer, Keocyt, AstraZeneca, BMS, Roche, Amgen, Merck, Shire, Hutchinson and Celgene.

## Funding

This work was supported by Novartis; Ipsen; the Liaison Committee between the Central Norway Regional Health Authority and the Norwegian University of Science and Technology (NTNU).

## Ethics

The work protocol was approved by ethics committees in Norway, Sweden and Denmark (Supplementary Methods). All patients signed informed written consent.

## Author contribution statement

H Sorbye and S Knappskog: shared last authorship.
