# Supplementary material for: The molecular characteristics of high-grade gastroenteropancreatic neuroendocrine neoplasms
Source: Endocr Relat Cancer. 2021 Oct 14;29(1):1–14. doi: 10.1530/ERC-21-0152 (PMC8630776; doi:10.1530/ERC-21-0152)
Supplement: Supplementary Methods [file supplementary_material.pdf]

Supplementary Methods

Patients and samples

Patients diagnosed with High Grade GEP NEN were from 2013 to 2017 included prospectively in a Nordic GEP Registry (n=279). Inclusion criteria were: histopathological confirmed diagnosis of neuroendocrine neoplasm (Ki-67>20%) with a gastroenteropancreatic primary or an unknown primary (CUP) predominantly with GI metastases (as defined by CT scans). Clinical information, tumour tissue and a whole blood sample for normal tissue analyses were collected. Histological sections (HE, CgA, synaptophycin, Ki-67) were collected and sent for centralized pathological re-evaluation by three pathologists (A.P., I.M.B.L. and A.C.) for validation of NEN G3 diagnosis, cell-type and recount of Ki-67. After initial independent evaluations, consensus was reached. Cases lacking normal-tissue (n=56), lacking slides for re-evaluation (n=2) or reassessed as NET G2 (n=1), adenocarcinoma (n=14), MiNen (n=23) or ambiguous (n=11) were excluded. Thus, a total of 181 samples were included for molecular analyses, out of which 152 were from patients with neuroendocrine carcinomas (NEC) and 29 with neuroendocrine tumours G3 (NET G3).

Tissue collection and isolation of DNA

For tumour samples, the areas of interest (i.e. areas with high tumour cell content), were visualized by morphology and synaptophysin, chromogranin A and Ki-67 staining. Tissue cores of 8 to 10 µM or (1.2 mm diameter) from tumour areas were manually macrodissected. The number of sections or cores taken from each block varied between the specimens, depending on the tumour tissue depth (in total, approximately 5 mg tissue was collected for each sample).

DNA isolation from formalin fixed paraffin embedded (FFPE) tumour specimens was carried out by Adaptive Focused Acoustics (AFA)-based extraction using the Covaris

truXTRAC FFPE DNA kit (Woburn, MA, USA). The extraction was performed according to the protocol provided by Covaris, with the following adjustments: the collected FFPE tissue, for each sample, were pooled in a screw-cap microTUBE. AFA was performed as per the manufacturer's instructions (protocol C) on a Covaris M220 Focused Ultrasonicator. Paraffin was removed and tissue rehydrated in a total amount of 100µl processing buffer master mix containing 88µl of tissue and SDS mixed with 22 µl of proteinase K (EC.3.4.21.64, Product No. SRE0005, Sigma–Aldrich). Homogenized tissue was then digested at 56 C overnight, followed by 1 h at 80 C to reverse the formaldehyde crosslinks. DNA was isolated from the digested lysates using columns of the Covaris truXTRAC FFPE DNA kit and was eluted in 100µl of Covaris BE buffer. Before library preparation for sequencing, all DNA samples underwent DNA repair to rectify some of the damage inflicted by fixation, paraffin embedding and isolation. For this purpose we used the DNA repair mix kit (NEBNext FFPE DNA kit, UK), according to the manufacturer's instructions.

Genomic DNA from normal tissue (blood) was isolated from peripheral blood using QIAamp DNA Mini Kit (Qiagen, Hilden, Germany), according to the manufacturer's protocol with the exception that we used 400 µl of blood as input in the isolation procedure.

MSI analysis

The microsatellite instability (MSI) status of each of the tumours was determined using the Promega MSI analysis system (Version 1.2, Promega, Madison, WI, US) following the manufacturer's instructions. Tumours with at least two of five mononucleotide markers altered were classified as MSI-H.

Quality control strategy

Before sequencing library was prepared, the DNA underwent several steps of quality control: 1) Preliminary quantification and purity by A_{260}/A_{230} and A_{260}/A_{280} ratios were assessed by measurements at a NanoDrop NT-1000 spectrophotometer. 2) The amount of dsDNA assessed by fluorometry using the Qubit double-stranded DNA (dsDNA) BR Assay Kit (Thermo Fisher Scientific, Waltham, MA, USA). 3) The size distribution / presence of high molecular weight DNA was assessed by Agilent 2100 Bioanalyzer (DNA 12000 kit; Agilent Technologies, Santa Clara, California, USA). For a randomly selected set of samples (n=26), we quantified the amount of amplifiable DNA by use of the KAPA hgDNA quantification and QC kit (Roche), as per manufacturer's instructions. This assessment relied on quantitative-PCR amplification of a 41 bp, 129 bp and 305 bp fragment of a highly conserved single copy gene. Real-time PCR was performed in a LightCycler 480 instrument (Roche, Basel, Switzerland) to generate standard curves. The 41 bp amplicon is used for absolute quantification of DNA samples against a set of DNA standards. DNA quality is assessed by normalizing the concentration obtained with the 129 and 305 bp amplicon against the one obtained with 41 bp assay (Q-score). Tumor DNA considered integer having optimally Q-score $\approx$ 1 for both Q129/Q41and the Q305/Q41 ratios.

Library preparation and sequencing

Targeted massive parallel sequencing was performed on DNA from FFPE tumor tissue and from matched normal peripheral blood DNA. DNA from each specimen was used to prepare Illumina libraries applying the Kapa Hyper Prep kit (Kapa Biosystem) and the Agilent SureSelect XT-kit (Agilent Technologies, Santa Clara, CA, United States of America). Targeted enrichment was performed using a custom RNA bait design according to the manufacturer's guidelines (SureSelect, Agilent, UK). The baits were targeted against an in-

house cancer gene panel designed to pull down entire coding regions of 360 cancer related genes (Yates, *et al.* 2015). The total design was of approximately 2 Mb in size.

We created targeted capture pulldown (average insert size, 140bp) libraries and generated paired-end sequence data (75bp) using MiSeq instrument (Illumina, San Diego, CA, United States of America) with an average sequencing depth of 136-fold (range 75x – 300x) for the tumours and 165-fold (range 50x- 272x) for the normal.

Data processing and bioinformatics analysis

Mapping and alteration calling

Raw sequence data was aligned to the human reference genome (Build-UCSC hg19) using BWA (Li and Durbin 2009) with default parameters. Quality control of the raw input data performed with FastQC program (http://www.bioinformatics.babraham.ac.uk/projects/fastqc). Somatic substitutions and insertions/deletions were detected using CaVEMan and Pindel respectively (Jones, *et al.* 2016); Raine, *et al.* 2015). ANNOVAR was used for vcf file annotation (Yang and Wang 2015). All somatic mutations were validated by manual inspection of sequencing reads using the Integrative Genomics Viewer IGV tool and COSMIC database. Further analysis was restricted to mutations in protein coding regions of the genome. In order to provide a complete overview of the mutations in the 360 genes in GEP-NEN, the data set was not restricted to driver-mutations. Allele specific copy number analysis was performed using FACETS (Fraction and Allele-Specific Copy Number Estimates from Tumor Sequencing) (Shen and Seshan 2016), suitable for targeted sequencing gene panels. FACETS was also applied for estimation of tumour ploidy as well as tumour purity in the tissue samples. Genomic Identifications of Significant Targets in Cancer (GISTIC) 2.0 (Mermel, *et al.* 2011) was used to identify frequent focal- and arm level-

amplifications and deletions (somatic copy number alterations; SCNAs). Arm-level gain and loss were defined as log2 depth ratios > 0.1 and < -0.1 respectively. Segments with 0 minor copy number were defined as LOH.

Illustrations

Oncoplots and illustrations of somatic mutation interactions (co-occurrence and mutual exclusivity) were generated by the R package Maftools (Mayakonda, *et al.* 2018). Focal levels of amplifications or deletions at a specific locus of the genome were illustrated using copy number R package (Nilsen, *et al.* 2012). IRanges R package as well as the Heatmap() function from ComplexHeatmap R package were utilized to build the heatmaps (Gu, *et al.* 2016); Lawrence, *et al.* 2013). Forest plots were generated by R package meta (Balduzzi, *et al.* 2019).

Targetable mutations

Genes affected by targetable mutations were defined based on literature search and classified in 12 different categories / functional pathways (BRAF, KRAS, MSI, FGFR, AKT, MTOR, PIK3CA, HER, Endocrine, TGFB, Homologous repair and DNA repair). The genes / mutations regarded as targetable within each category are listed in Supplementary Table S1. The specific alterations of oncogenes termed "targetable" were restricted those where drugs have been proven to have an effect; e.g. for the BRAF and KRAS genes, the term "targetable alteration" was restricted to *BRAF* V600E/K and *KRAS* G12C, respectively. For tumour suppressor genes (typically those involved in DNA repair), any alteration potentially impairing function was counted as targetable; the lists for tumour suppressor genes involved in homologous repair and general DNA repair, was previously published as list used for identification of predictive markers for PARP-inhibition (olaparib) in primary treatment naïve

breast cancer (Eikesdal, *et al.* 2020). Tumours scored as MSI were included as harboring targetable alterations, as MSI is increasingly used as a biomarker for checkpoint inhibitors.

Pathway analyses

Pathway analyses was performed using a restricted number of known oncogenes and tumor suppressor genes involved in key oncogenic signaling pathways (Supplementary Table S1). The schematic representation was done by the function oncoPrint() from ComplexHeatmap R package (Gu, *et al.* 2016).

Prediction model

A prediction model for classification of tumours into the categories LC NEC or NET G3 was built, based on mutational status of nine genes (*APC*, *ATRX*, *BRAF*, *DAXX*, *KRAS*, *MEN1*, *MYO5B*, *SMAD2* and *TP53*). Classification was performed using C5.0 decision tree algorithm implemented in R package C50 (v0.1.2) (Quinlan 2007). Thirty boosting iterations were used. Input data and code used are given in Supplementary files S1-S3.

Statistics

All statistical analyses were performed in the statistical programming language R (v3.5.1). Differences in mutation frequency between groups were assessed by odds ratio (OR) estimates with 95% confidence intervals (CI) and by Fischer exact test. Overall survival (OS) was assessed from the date of diagnosis to the date of death or last follow-up. Survival curves were drawn according the Kaplan-Meier method and differences within groups were assessed by long-rank tests. All p-values are given as two sided and p-values < 0.05 were considered statistically significant.

Ethics

The research protocol was approved by ethics committees in Norway (REK vest 2012/940), Sweden (REC Uppsala Dnr 2012/285) and Denmark (Region Hovedstaden H-4-2012-108). All patients signed informed written consent.

Supplementary references

Balduzzi S, Rucker G & Schwarzer G 2019 How to perform a meta-analysis with R: a practical tutorial. *Evid Based Ment Health* 22 153-160.

Eikesdal HP, Yndestad S, Elzawahry A, Llop-Guevara A, Gilje B, Blix ES, Espelid H, Lundgren S, Geisler J, Vagstad G, et al. 2020 Olaparib monotherapy as primary treatment in unselected triple negative breast cancer. *Ann Oncol*.

Gu Z, Eils R & Schlesner M 2016 Complex heatmaps reveal patterns and correlations in multidimensional genomic data. *Bioinformatics* 32 2847-2849.

Jones D, Raine KM, Davies H, Tarpey PS, Butler AP, Teague JW, Nik-Zainal S & Campbell PJ 2016 cgpCaVEManWrapper: Simple Execution of CaVEMan in Order to Detect Somatic Single Nucleotide Variants in NGS Data. *Curr Protoc Bioinformatics* 56 15 10 11-15 10 18.

Lawrence M, Huber W, Pages H, Aboyoun P, Carlson M, Gentleman R, Morgan MT & Carey VJ 2013 Software for computing and annotating genomic ranges. *PLoS Comput Biol* 9 e1003118.

Li H & Durbin R 2009 Fast and accurate short read alignment with Burrows-Wheeler transform. *Bioinformatics* 25 1754-1760.

Mayakonda A, Lin DC, Assenov Y, Plass C & Koeffler HP 2018 Maftools: efficient and comprehensive analysis of somatic variants in cancer. *Genome Res* 28 1747-1756.

Mermel CH, Schumacher SE, Hill B, Meyerson ML, Beroukhim R & Getz G 2011 GISTIC2.0 facilitates sensitive and confident localization of the targets of focal somatic copy-number alteration in human cancers. *Genome Biol* 12 R41.

Nilsen G, Liestol K, Van Loo P, Moen Vollan HK, Eide MB, Rueda OM, Chin SF, Russell R, Baumbusch LO, Caldas C, et al. 2012 Copynumber: Efficient algorithms for single- and multi-track copy number segmentation. *BMC Genomics* 13 591.

Quinlan JR 2007 C5. http://rulequest.com.

Raine KM, Hinton J, Butler AP, Teague JW, Davies H, Tarpey P, Nik-Zainal S & Campbell PJ 2015 cgpPindel: Identifying Somatically Acquired Insertion and Deletion Events from Paired End Sequencing. *Curr Protoc Bioinformatics* 52 15 17 11-12.

Shen R & Seshan VE 2016 FACETS: allele-specific copy number and clonal heterogeneity analysis tool for high-throughput DNA sequencing. *Nucleic Acids Res* 44 e131.

Yang H & Wang K 2015 Genomic variant annotation and prioritization with ANNOVAR and wANNOVAR. *Nat Protoc* 10 1556-1566.

Yates LR, Gerstung M, Knappskog S, Desmedt C, Gundem G, Van Loo P, Aas T, Alexandrov LB, Larsimont D, Davies H, et al. 2015 Subclonal diversification of primary breast cancer revealed by multiregion sequencing. *Nat Med* 21 751-759.
