# Supplementary figures and images for: The molecular characteristics of high-grade gastroenteropancreatic neuroendocrine neoplasms
Source: Endocr Relat Cancer. 2021 Oct 14;29(1):1–14. doi: 10.1530/ERC-21-0152 (PMC8630776; doi:10.1530/ERC-21-0152)

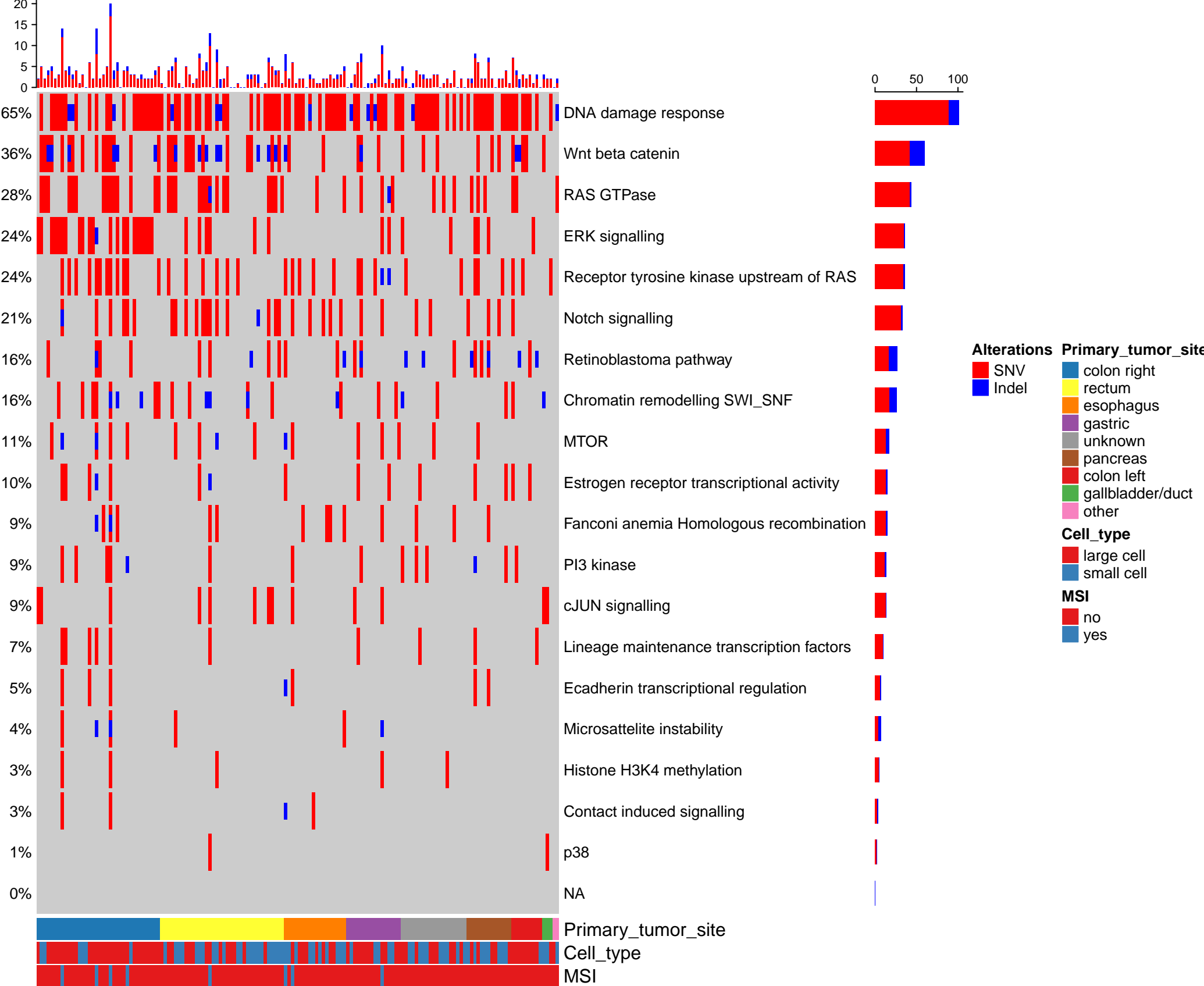

Supplement: Suppl. Figure 1. Pathway analysis of the NEC cohort. Coloured squares indicate genetic alterations, i.e single nucleotide variants (SNVs; red) and insertions/deletions (InDels;blue). Columns represent patients and rows represent pathways. Percentage referring to the fractions of patients affected by [file supplementary_figure_1.pdf]

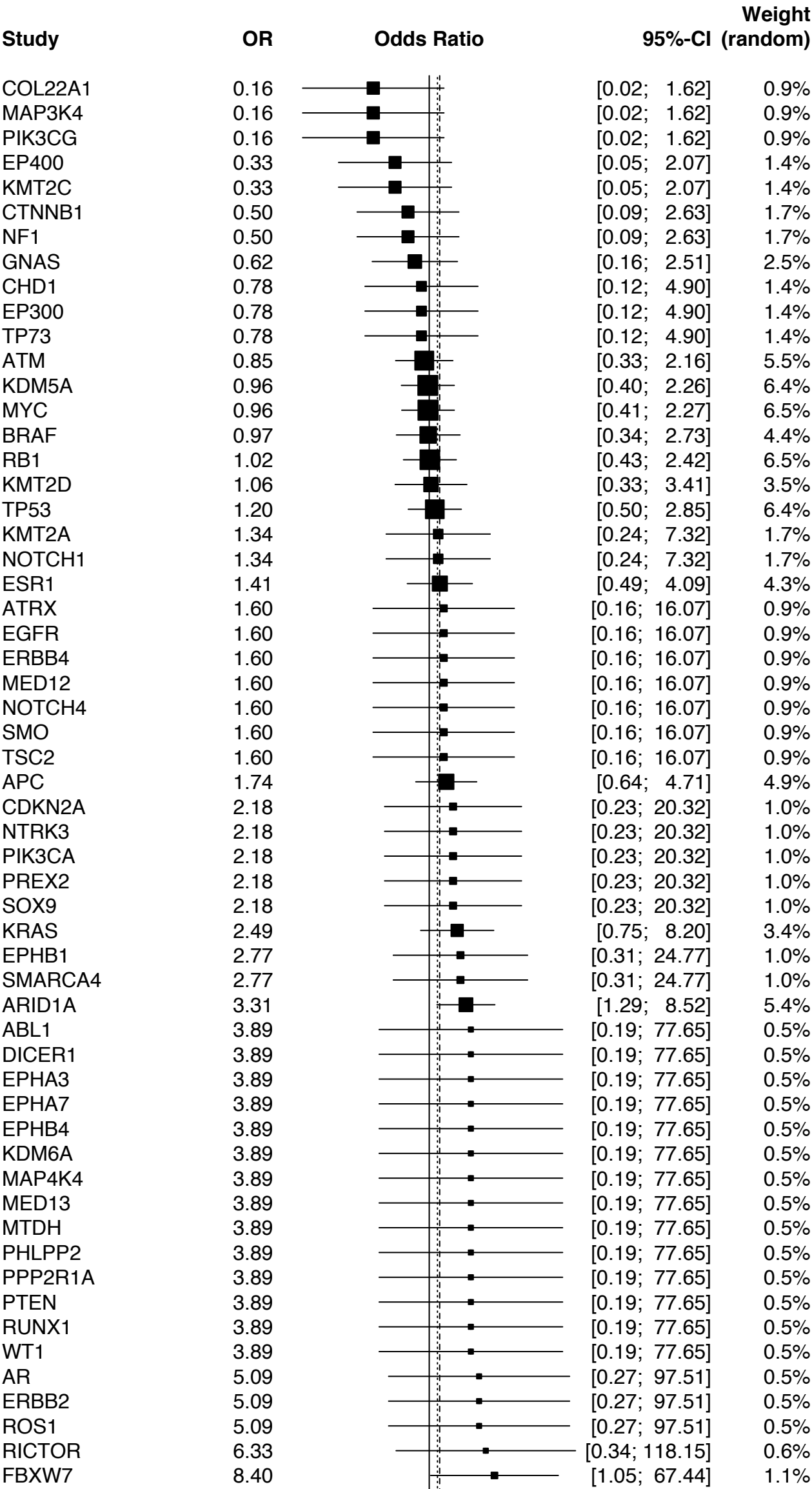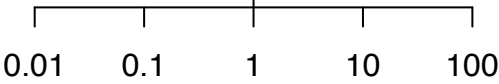

Supplement: Suppl Figure 3. Forest plot showing the enrichments for altered genes in smokers versus non-smokers among NEC patients (illustrated as odds ratio [OR] where OR<1 indicates enrichment in smokers and OR>1 indicates enrichment in non-smokers). The plot incudes all genes mutated in minimum of 3 of the p [file supplementary_figure_3.pdf]

A

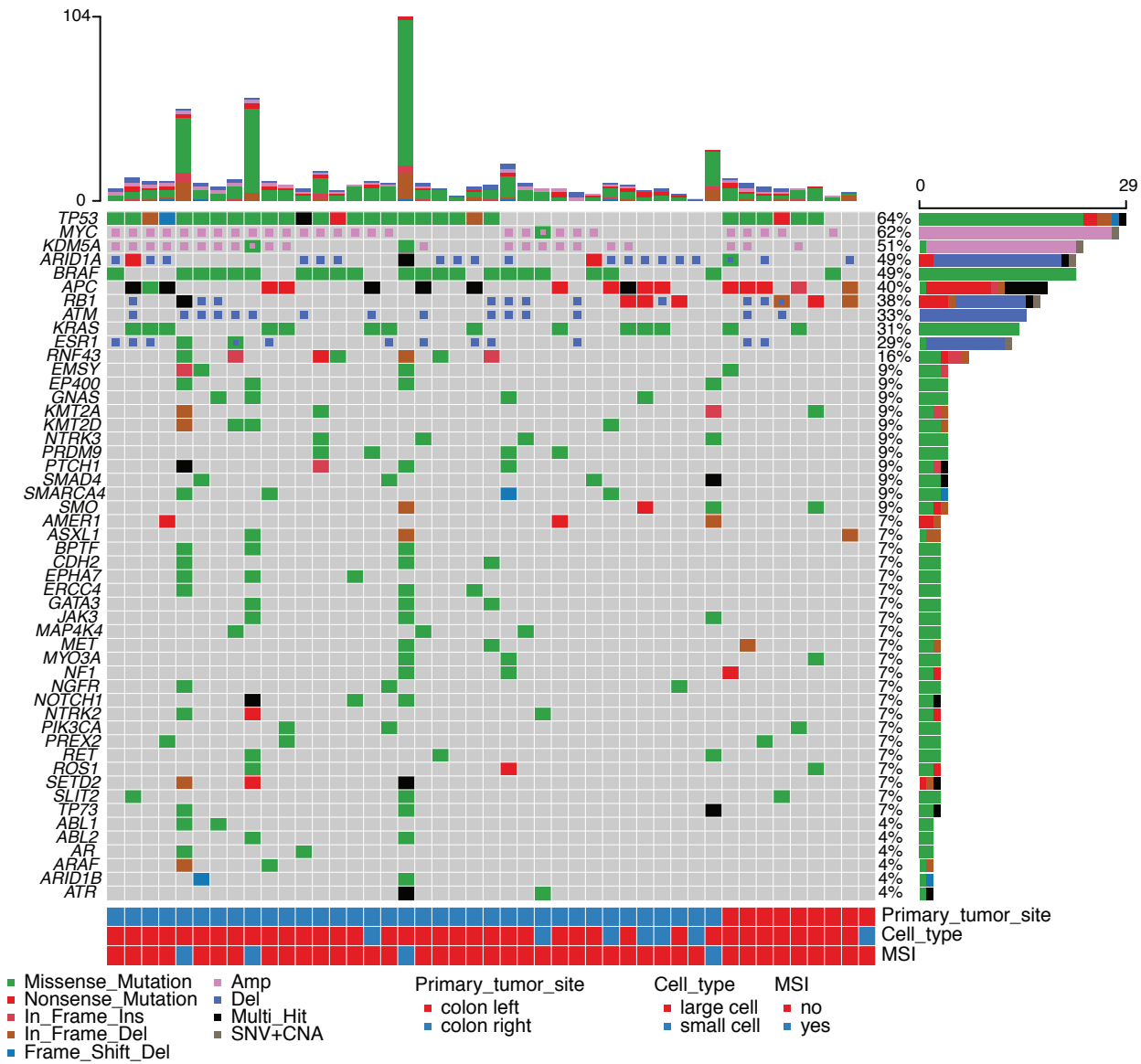

B

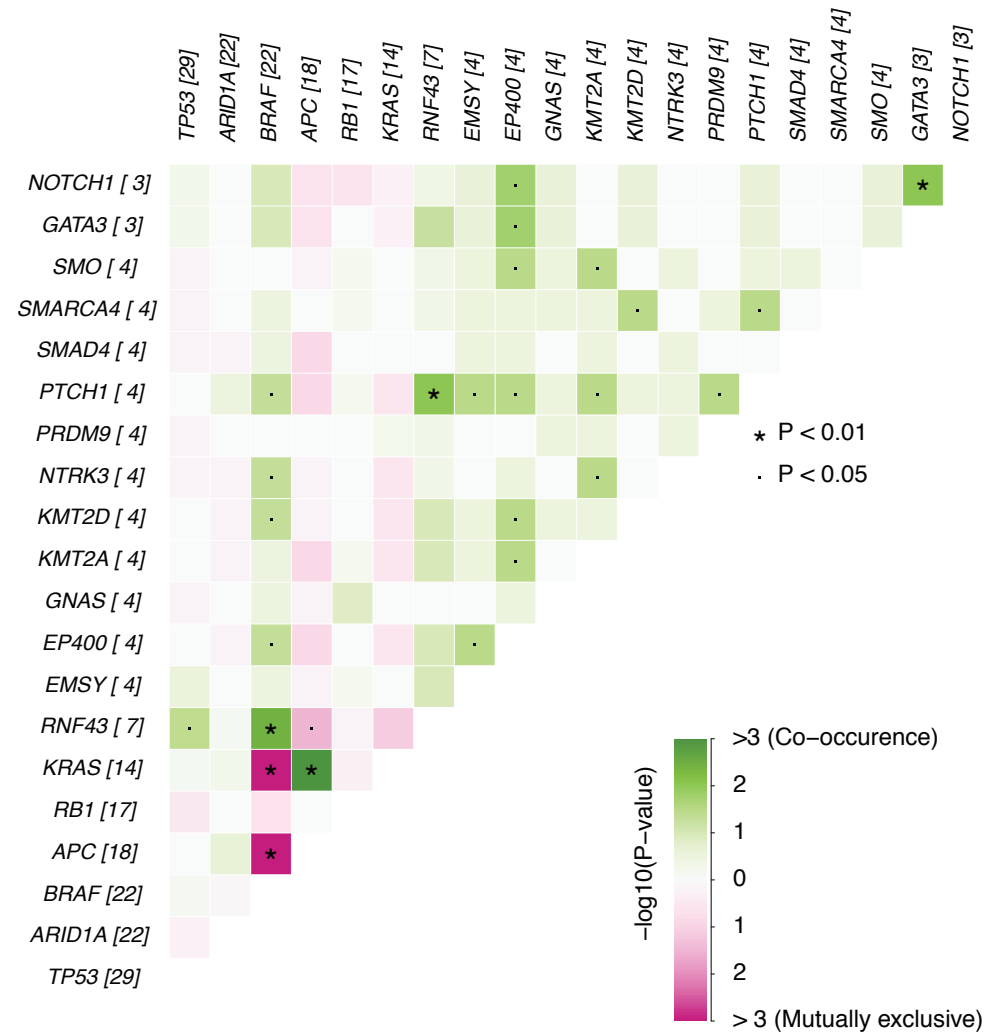

Supplement: Suppl Figure 4. A) Oncoplot showing the top 50 most frequently altered genes (rows) among 45 colonic NEC primaries (columns). Upper panel shows the mutational burden per sample. Percentages on the right represent mutations frequency per gene. The panel under the oncoplot area is composed of 3 single [file supplementary_figure_4.pdf]

A

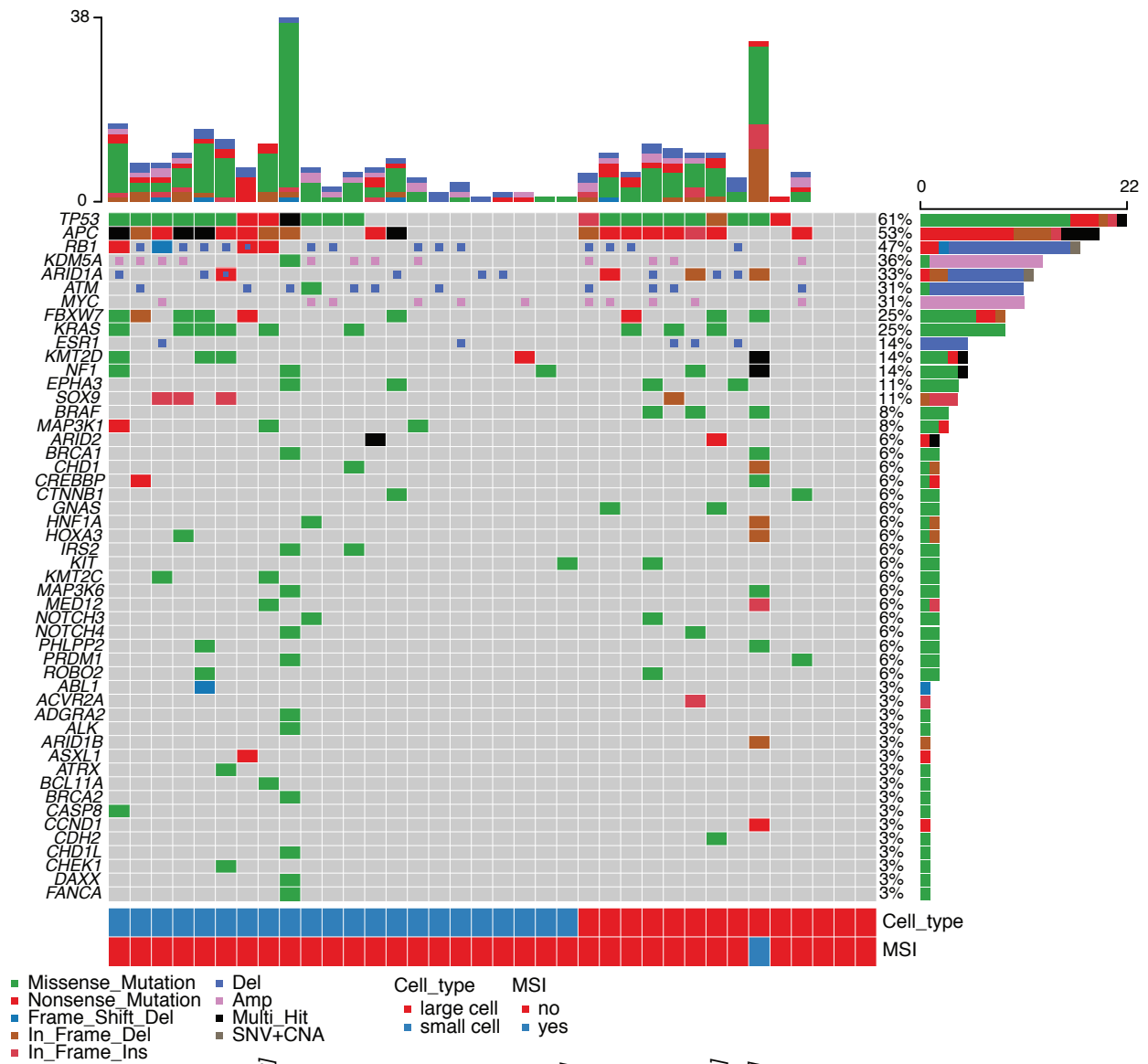

B

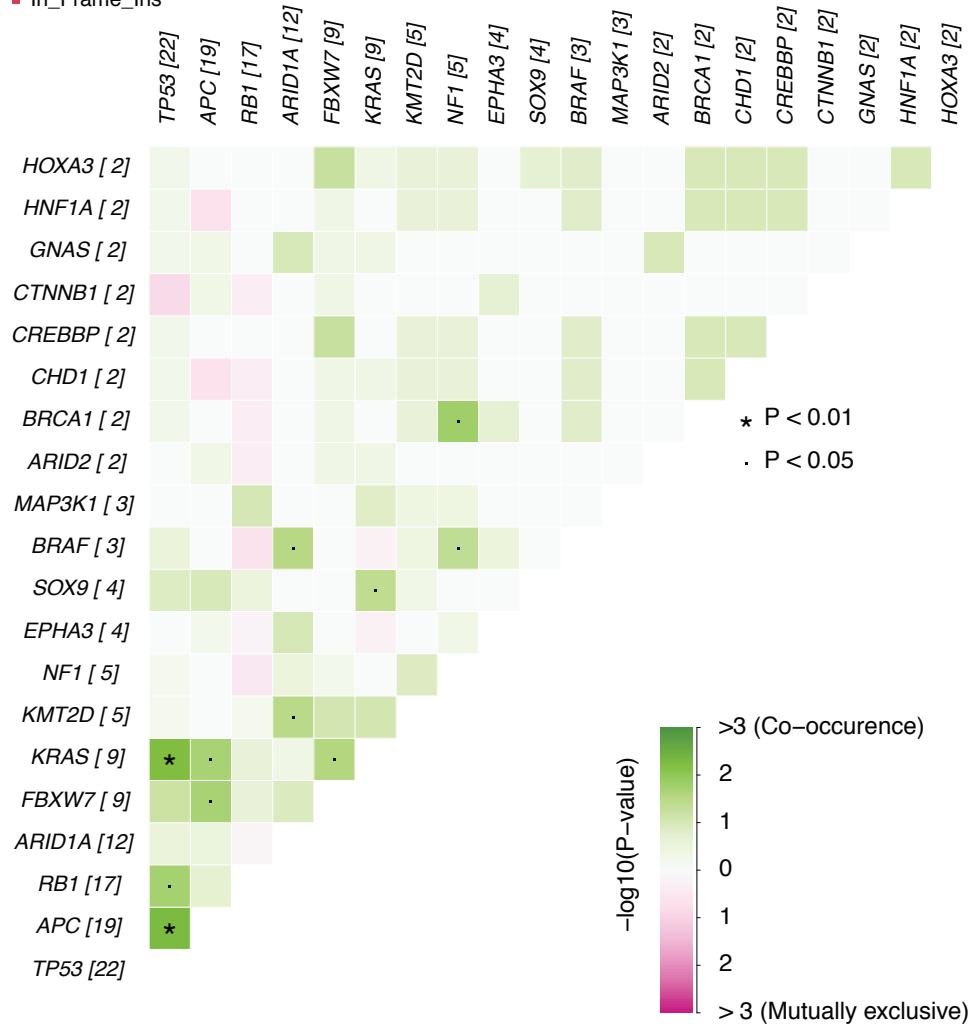

Supplement: Suppl Figure 5. A) Oncoplot showing the top 50 most frequently altered genes (rows) among 36 rectal NEC primaries (columns). Upper panel shows the mutational burden per sample. Percentages on the right represent mutations frequency per gene. The panel under the oncoplot area is composed of 3 single  [file supplementary_figure_5.pdf]

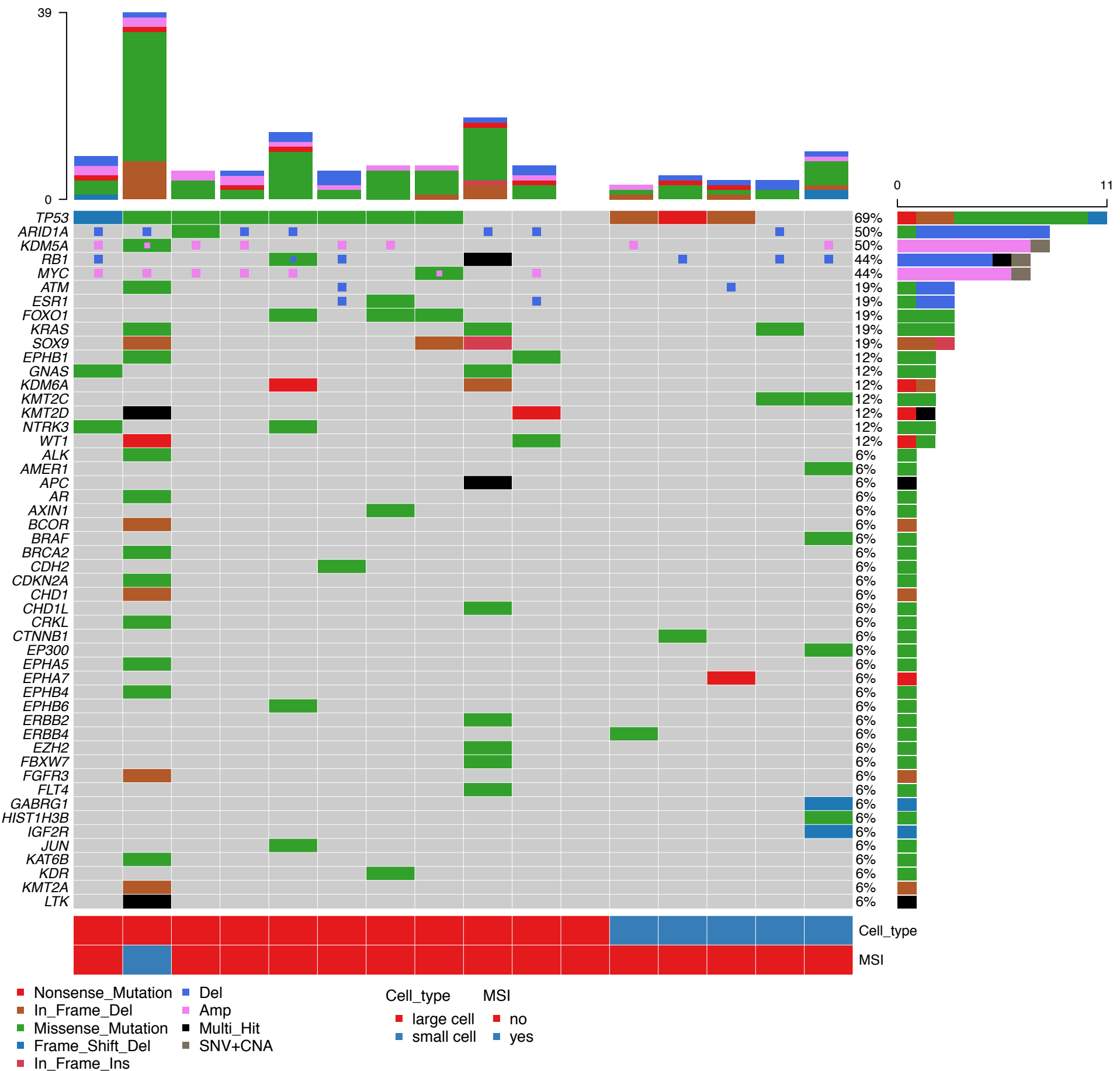

Supplement: Suppl Figure 6. Oncoplot showing the top 50 most frequently altered genes (rows) among 16 gastric NEC primaries (columns). Upper panel shows the mutational burden per sample. Percentages on the right represent mutations frequency per gene. The panel under the oncoplot area is composed of 3 single ro [file supplementary_figure_6.pdf]

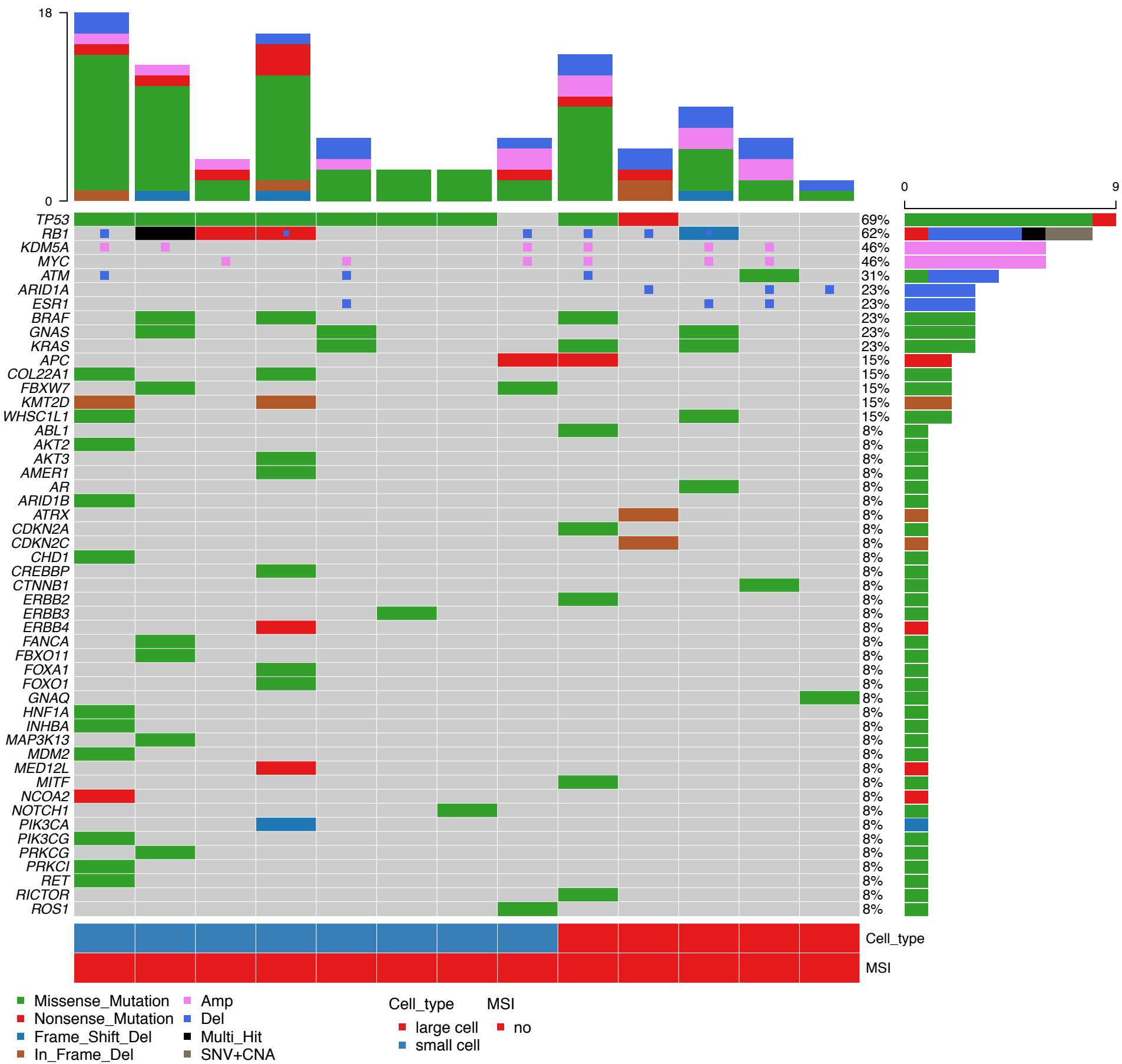

Supplement: Suppl Figure 7. Oncoplot showing the top 50 most frequently altered genes (rows) among 13 pancreatic NEC primaries (columns). Upper panel shows the mutational burden per sample. Percentages on the right represent mutations frequency per gene. The panel under the oncoplot area is composed of 3 single [file supplementary_figure_7.pdf]

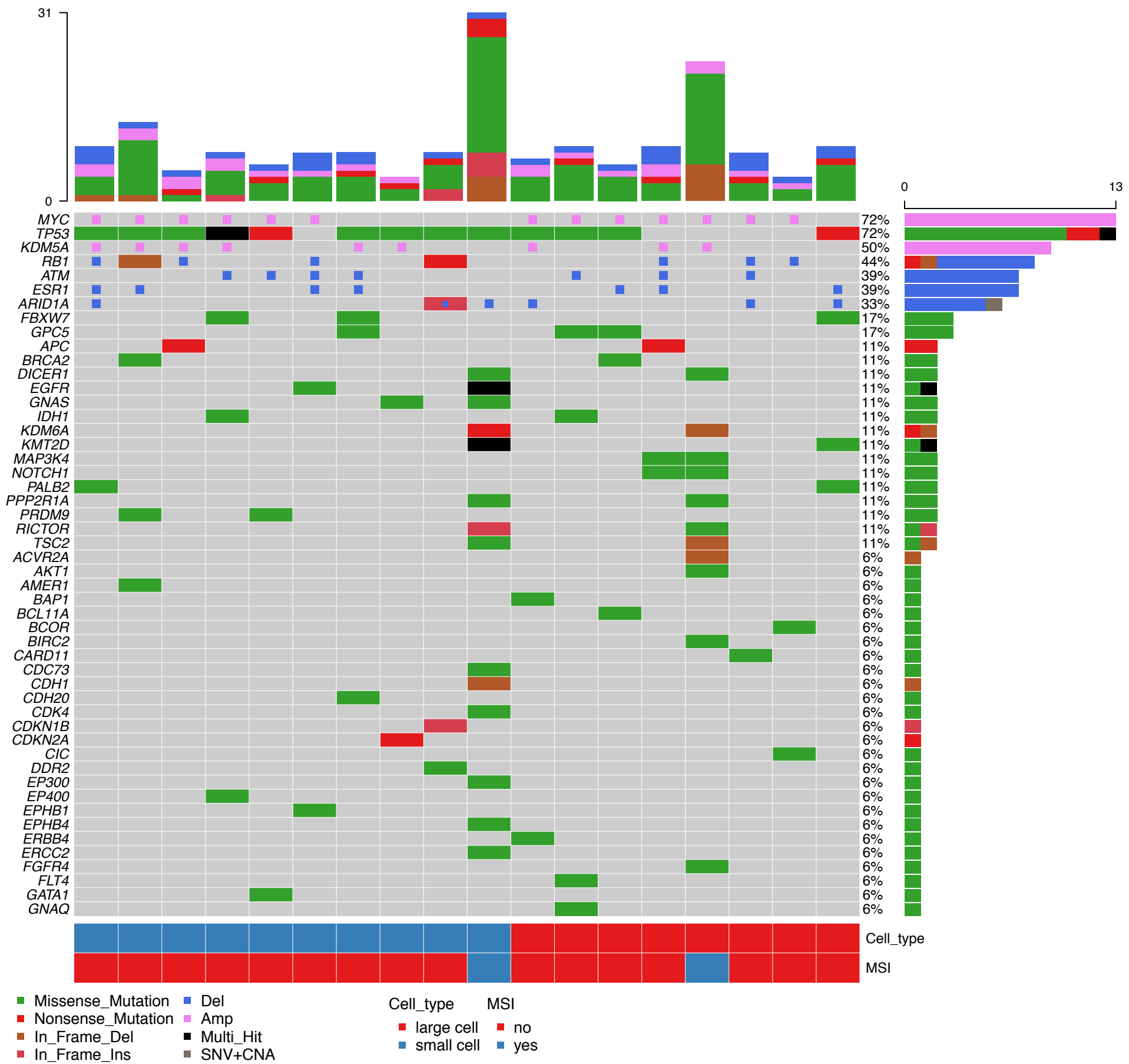

Supplement: Suppl Figure 8. Oncoplot showing the top 50 most frequently altered genes (rows) among 18 esophageal NEC primaries (columns). Upper panel shows the mutational burden per sample. Percentages on the right represent mutations frequency per gene. The panel under the oncoplot area is composed of 3 single [file supplementary_figure_8.pdf]

**A**

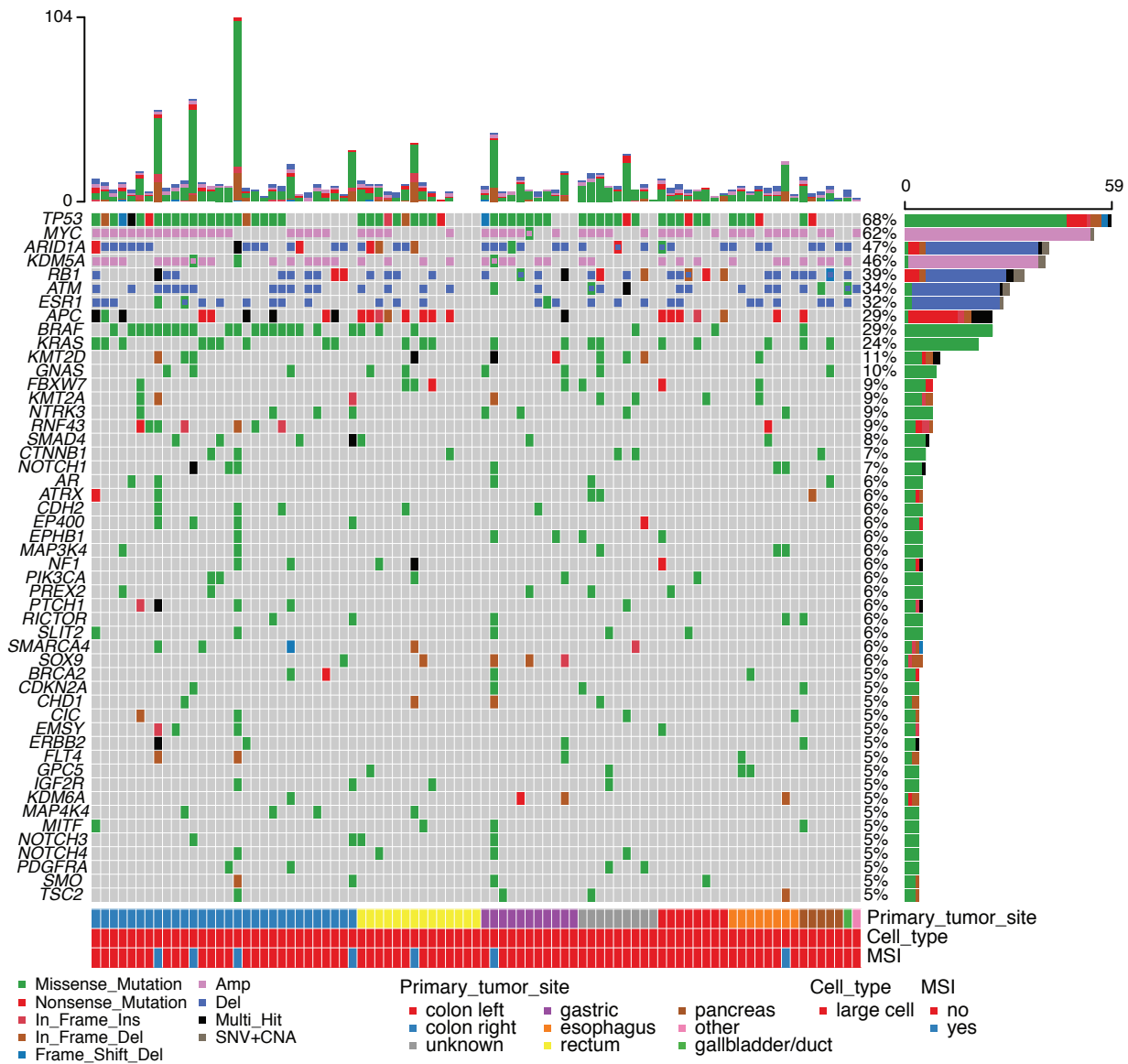

# B

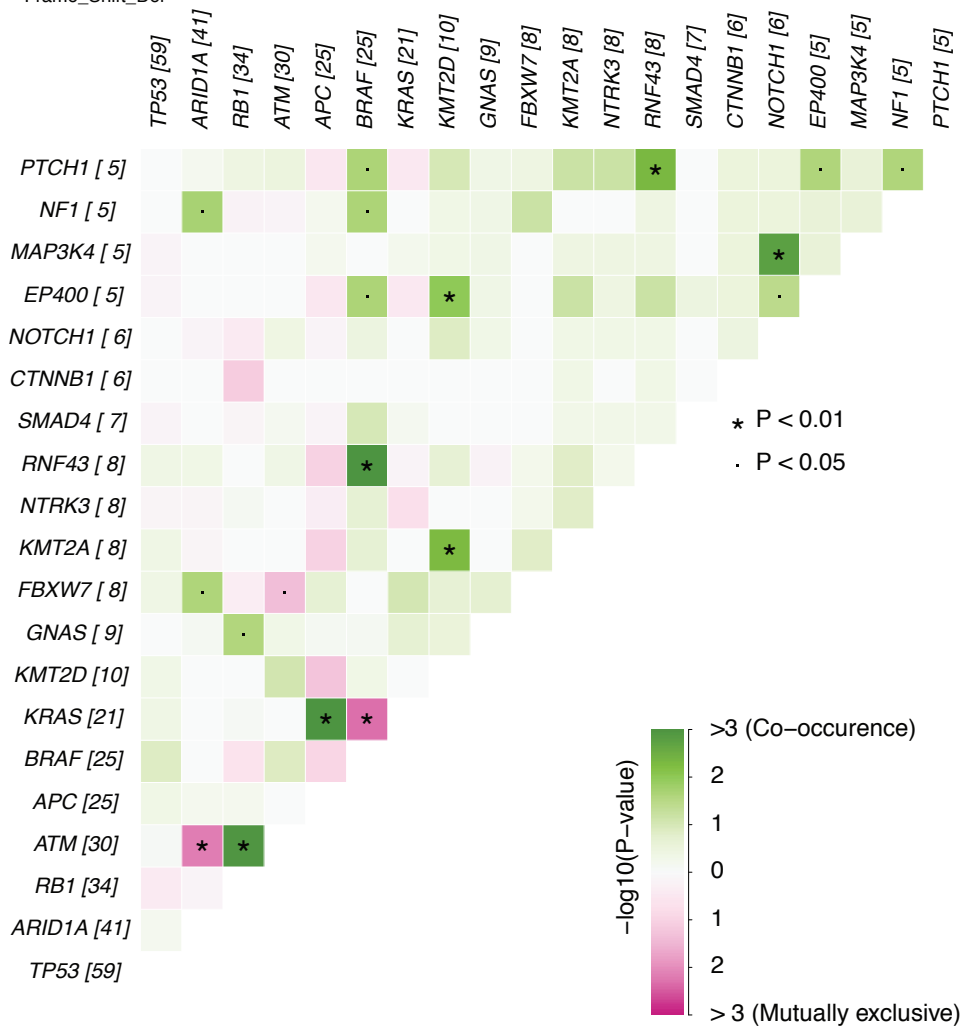

Supplement: Suppl Figure 9. A) Oncoplot showing the top 50 most frequently altered genes (rows) among 87 large cell NEC patients (columns). Upper panel shows the mutational burden per sample. Percentages on the right represent mutations frequency per gene. The panel under the oncoplot area is composed of 3 sing [file supplementary_figure_9.pdf]

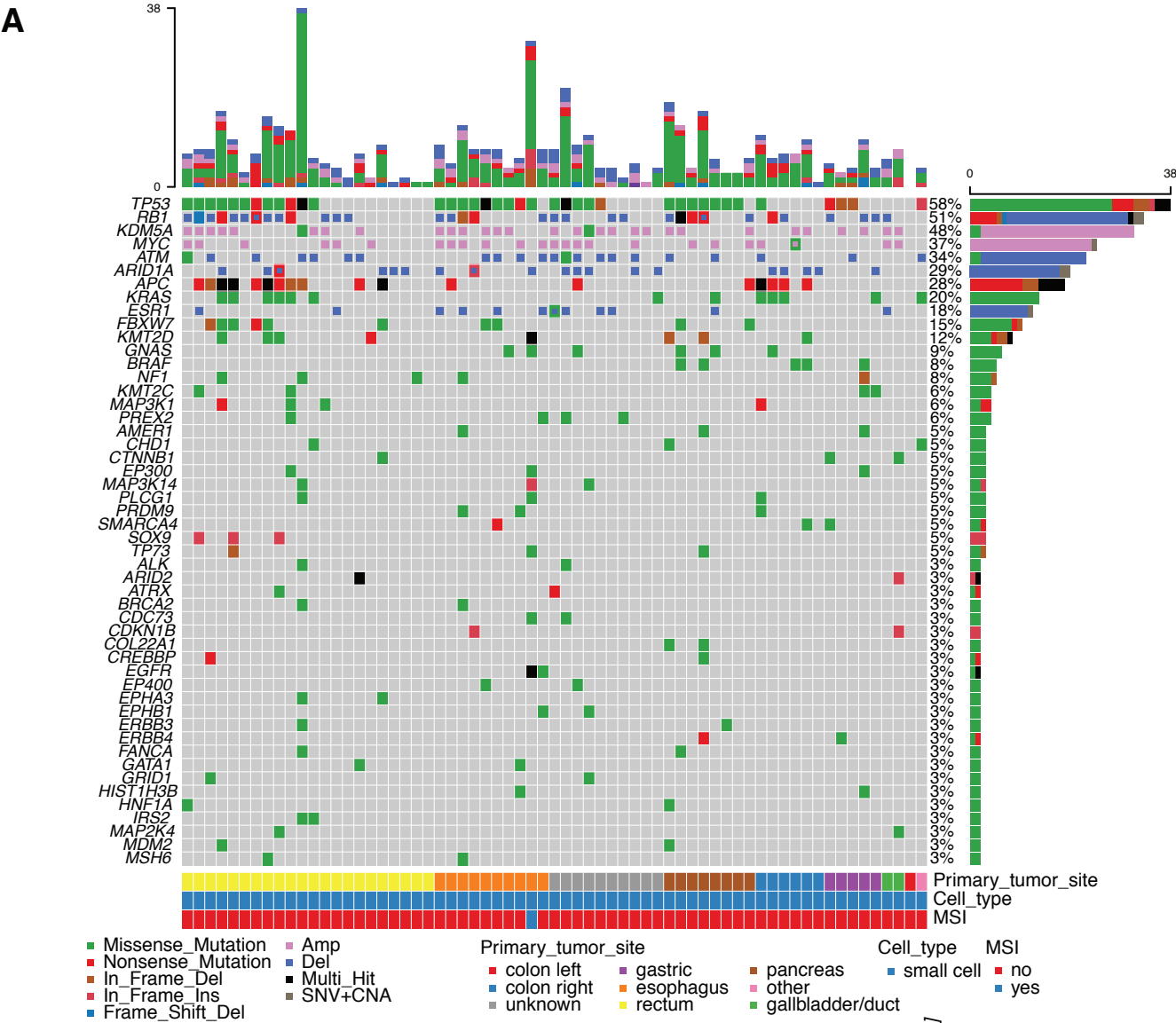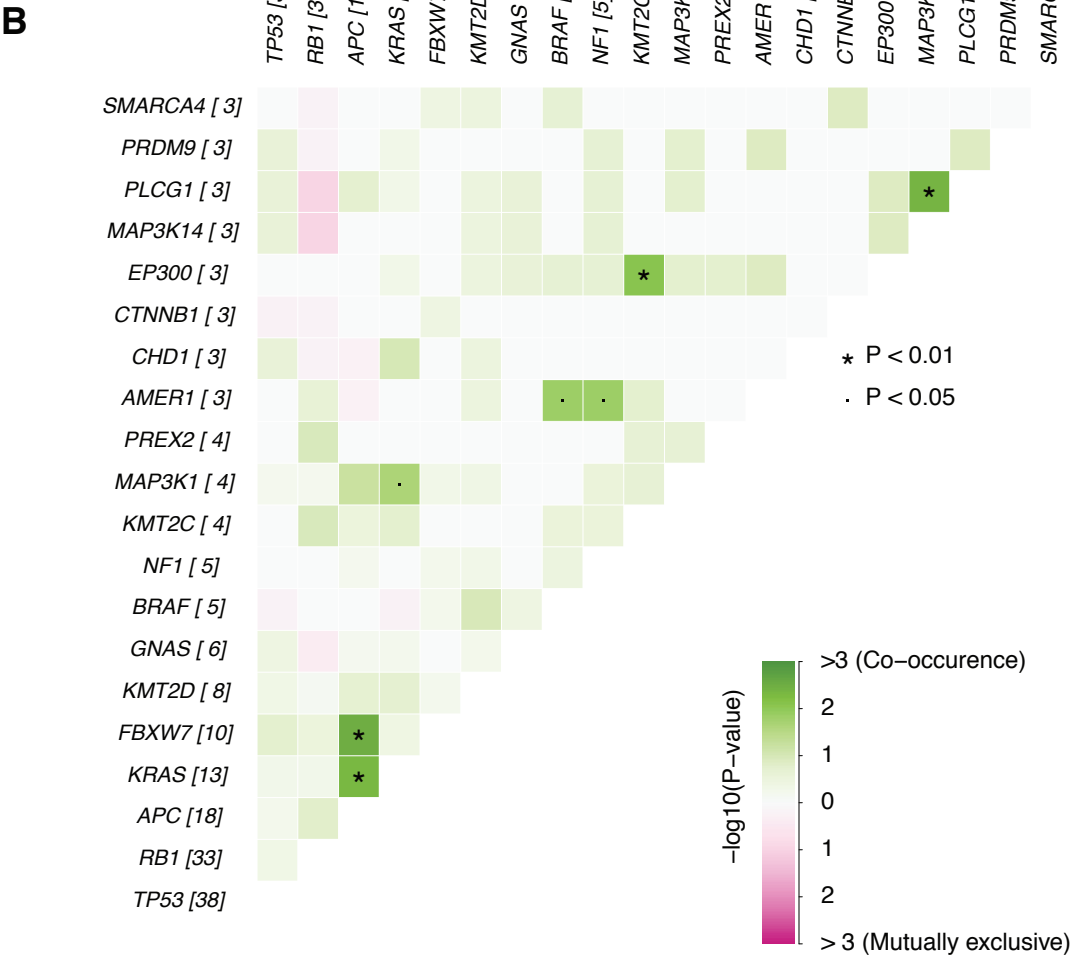

Supplement: Suppl Figure 10. A) Oncoplot showing the top 50 most frequently altered genes (rows) among 65 small cell NEC patients (columns). Upper panel shows the mutational burden per sample. Percentages on the right represent mutations frequency per gene. The panel under the oncoplot area is composed of 3 sin [file supplementary_figure_10.pdf]

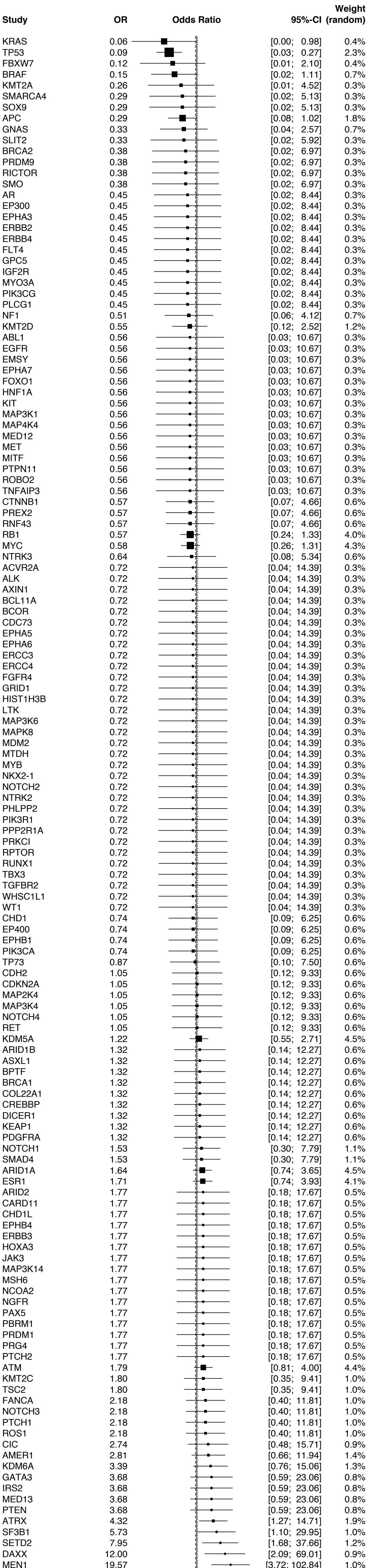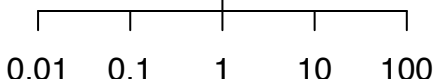

Supplement: Suppl. Figure 13. Forest plot showing the enrichments for altered genes patients with NEC versus NET G3 (illustrated as odds ratio [OR] where OR<1 indicates enrichment in NEC and OR>1 indicates enrichment in NET G3). The plot incudes all genes mutated in minimum of 3 of the patients. [file supplementary_figure_13.pdf]

**A**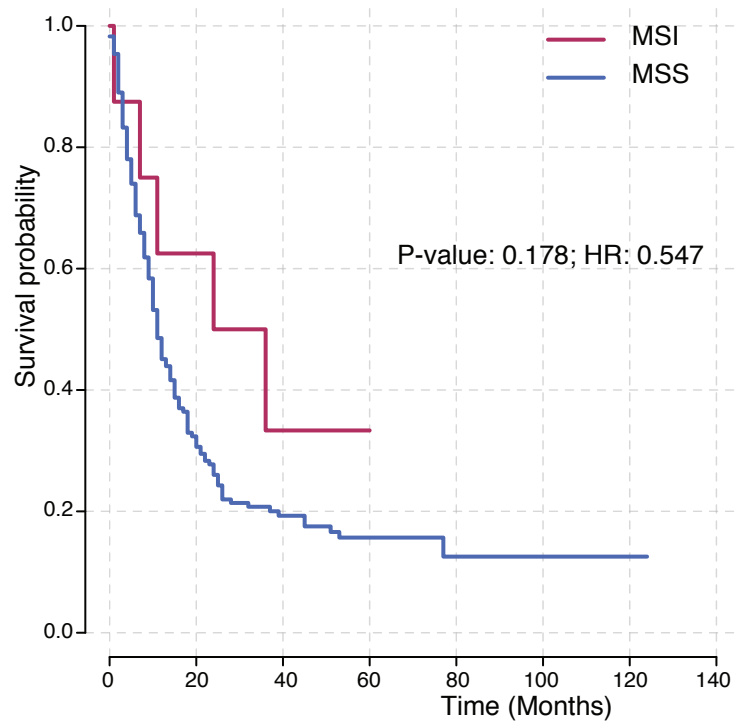**B**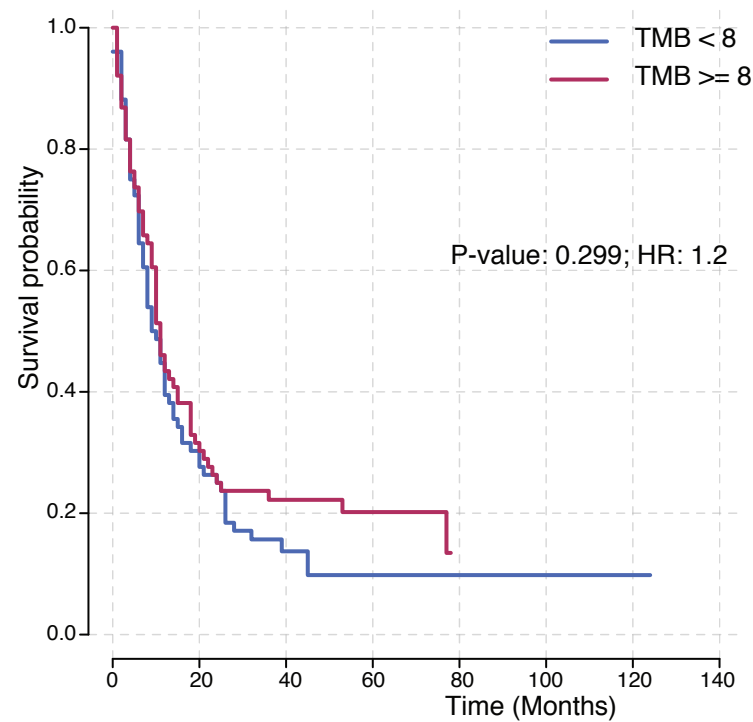**C**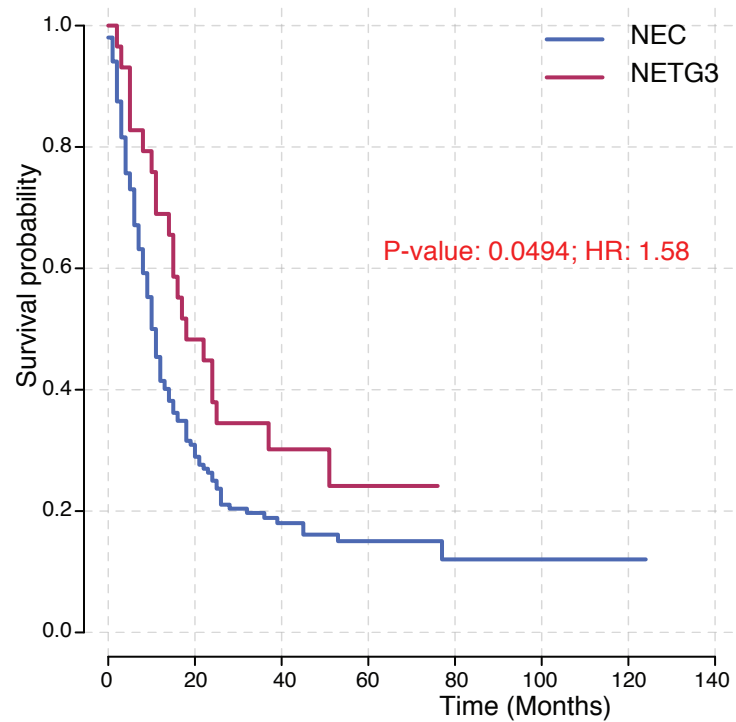**D**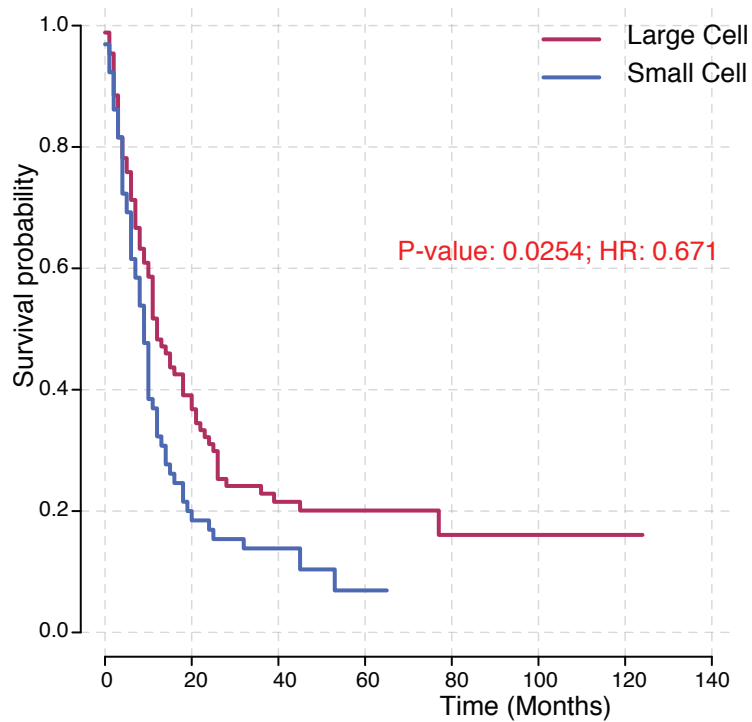

Supplement: Suppl. Figure 15. Kaplan-Meier overall survival analyses of A) NEC patients with MSI versus MSS, B) NEC according to mutational burden (i.e. number of mutations and alterations within the analyzed 360 cancer genes), below vs. above median (<8 vs. >=8), C) NEC versus NETG3, D) Large cell versus Small [file supplementary_figure_15.pdf]
