# Supplementary material for: The molecular characteristics of high-grade gastroenteropancreatic neuroendocrine neoplasms
Source: Endocr Relat Cancer. 2021 Oct 14;29(1):1–14. doi: 10.1530/ERC-21-0152 (PMC8630776; doi:10.1530/ERC-21-0152)

Study	OR	95%-CI	Weight (random)
MYC	0.17	[0.04; 0.80]	1.9%
AMER1	0.17	[0.03; 1.06]	1.4%
ABL1	0.28	[0.03; 2.94]	0.8%
EP300	0.28	[0.03; 2.94]	0.8%
KMT2C	0.28	[0.03; 2.94]	0.8%
MITF	0.28	[0.03; 2.94]	0.8%
GNAS	0.31	[0.07; 1.29]	2.3%
KDM5A	0.36	[0.10; 1.22]	3.1%
ESR1	0.38	[0.12; 1.22]	3.4%
GPC5	0.38	[0.04; 3.69]	0.9%
IGF2R	0.38	[0.04; 3.69]	0.9%
MAP2K4	0.38	[0.04; 3.69]	0.9%
MAP3K4	0.38	[0.04; 3.69]	0.9%
APC	0.40	[0.12; 1.27]	3.4%
RB1	0.44	[0.14; 1.43]	3.4%
ATRX	0.48	[0.05; 4.45]	0.9%
BRCA2	0.48	[0.05; 4.45]	0.9%
PRDM9	0.48	[0.05; 4.45]	0.9%
NOTCH1	0.58	[0.06; 5.23]	1.0%
PREX2	0.58	[0.06; 5.23]	1.0%
SLIT2	0.58	[0.06; 5.23]	1.0%
KRAS	0.59	[0.17; 2.06]	2.9%
CTNNB1	0.68	[0.08; 6.03]	1.0%
ARID1A	0.73	[0.23; 2.29]	3.5%
ACVR2A	0.74	[0.04; 15.12]	0.5%
ALK	0.74	[0.04; 15.12]	0.5%
ASXL1	0.74	[0.04; 15.12]	0.5%
AXIN1	0.74	[0.04; 15.12]	0.5%
BCOR	0.74	[0.04; 15.12]	0.5%
CARD11	0.74	[0.04; 15.12]	0.5%
CDC73	0.74	[0.04; 15.12]	0.5%
CHD1L	0.74	[0.04; 15.12]	0.5%
CREBBP	0.74	[0.04; 15.12]	0.5%
EPHA5	0.74	[0.04; 15.12]	0.5%
EPHB4	0.74	[0.04; 15.12]	0.5%
ERBB3	0.74	[0.04; 15.12]	0.5%
ERCC4	0.74	[0.04; 15.12]	0.5%
FGFR4	0.74	[0.04; 15.12]	0.5%
GATA3	0.74	[0.04; 15.12]	0.5%
GRID1	0.74	[0.04; 15.12]	0.5%
HOXA3	0.74	[0.04; 15.12]	0.5%
IRS2	0.74	[0.04; 15.12]	0.5%
JAK3	0.74	[0.04; 15.12]	0.5%
LTK	0.74	[0.04; 15.12]	0.5%
MAP3K1	0.74	[0.04; 15.12]	0.5%
MAP3K14	0.74	[0.04; 15.12]	0.5%
MAP3K6	0.74	[0.04; 15.12]	0.5%
MAPK8	0.74	[0.04; 15.12]	0.5%
MDM2	0.74	[0.04; 15.12]	0.5%
MED12	0.74	[0.04; 15.12]	0.5%
MED13	0.74	[0.04; 15.12]	0.5%
MSH6	0.74	[0.04; 15.12]	0.5%
MTDH	0.74	[0.04; 15.12]	0.5%
MYB	0.74	[0.04; 15.12]	0.5%
NCOA2	0.74	[0.04; 15.12]	0.5%
NGFR	0.74	[0.04; 15.12]	0.5%
NKX2-1	0.74	[0.04; 15.12]	0.5%
NTRK2	0.74	[0.04; 15.12]	0.5%
PBRM1	0.74	[0.04; 15.12]	0.5%
PHLPP2	0.74	[0.04; 15.12]	0.5%
PIK3R1	0.74	[0.04; 15.12]	0.5%
PPP2R1A	0.74	[0.04; 15.12]	0.5%
PRDM1	0.74	[0.04; 15.12]	0.5%
PRKCI	0.74	[0.04; 15.12]	0.5%
PTCH2	0.74	[0.04; 15.12]	0.5%
PTEN	0.74	[0.04; 15.12]	0.5%
PTPN11	0.74	[0.04; 15.12]	0.5%
RUNX1	0.74	[0.04; 15.12]	0.5%
SETD2	0.74	[0.04; 15.12]	0.5%
TBX3	0.74	[0.04; 15.12]	0.5%
TGFBR2	0.74	[0.04; 15.12]	0.5%
WT1	0.74	[0.04; 15.12]	0.5%
ATM	0.85	[0.26; 2.74]	3.3%
BRAF	0.87	[0.22; 3.40]	2.5%
NF1	0.89	[0.10; 7.66]	1.0%
ARID1B	0.96	[0.05; 18.82]	0.5%
BPTF	0.96	[0.05; 18.82]	0.5%
BRCA1	0.96	[0.05; 18.82]	0.5%
CDKN2A	0.96	[0.05; 18.82]	0.5%
CIC	0.96	[0.05; 18.82]	0.5%
COL22A1	0.96	[0.05; 18.82]	0.5%
DICER1	0.96	[0.05; 18.82]	0.5%
EGFR	0.96	[0.05; 18.82]	0.5%
EMSY	0.96	[0.05; 18.82]	0.5%
EPHA7	0.96	[0.05; 18.82]	0.5%
FOXO1	0.96	[0.05; 18.82]	0.5%
HNF1A	0.96	[0.05; 18.82]	0.5%
KEAP1	0.96	[0.05; 18.82]	0.5%
KIT	0.96	[0.05; 18.82]	0.5%
MAP4K4	0.96	[0.05; 18.82]	0.5%
MET	0.96	[0.05; 18.82]	0.5%
PDGFRA	0.96	[0.05; 18.82]	0.5%
ROBO2	0.96	[0.05; 18.82]	0.5%
TNFAIP3	0.96	[0.05; 18.82]	0.5%
CDH2	1.18	[0.06; 22.58]	0.5%
EPHA3	1.18	[0.06; 22.58]	0.5%
ERBB2	1.18	[0.06; 22.58]	0.5%
ERBB4	1.18	[0.06; 22.58]	0.5%
FANCA	1.18	[0.06; 22.58]	0.5%
FLT4	1.18	[0.06; 22.58]	0.5%
KDM6A	1.18	[0.06; 22.58]	0.5%
MYO3A	1.18	[0.06; 22.58]	0.5%
NOTCH3	1.18	[0.06; 22.58]	0.5%
NOTCH4	1.18	[0.06; 22.58]	0.5%
PIK3CG	1.18	[0.06; 22.58]	0.5%
PLCG1	1.18	[0.06; 22.58]	0.5%
PTCH1	1.18	[0.06; 22.58]	0.5%
RET	1.18	[0.06; 22.58]	0.5%
ROS1	1.18	[0.06; 22.58]	0.5%
SMO	1.18	[0.06; 22.58]	0.5%
TSC2	1.18	[0.06; 22.58]	0.5%
EP400	1.41	[0.08; 26.42]	0.5%
EPHB1	1.41	[0.08; 26.42]	0.5%
RICTOR	1.41	[0.08; 26.42]	0.5%
TP73	1.41	[0.08; 26.42]	0.5%
CHD1	1.64	[0.09; 30.32]	0.5%
PIK3CA	1.64	[0.09; 30.32]	0.5%
SMAD4	1.64	[0.09; 30.32]	0.5%
NTRK3	1.87	[0.10; 34.29]	0.5%
SMARCA4	1.87	[0.10; 34.29]	0.5%
SOX9	1.87	[0.10; 34.29]	0.5%
KMT2A	2.11	[0.12; 38.32]	0.5%
RNF43	2.11	[0.12; 38.32]	0.5%
TP53	2.20	[0.70; 6.95]	3.5%
FBXW7	3.89	[0.22; 68.59]	0.6%
KMT2D	4.44	[0.25; 77.93]	0.6%

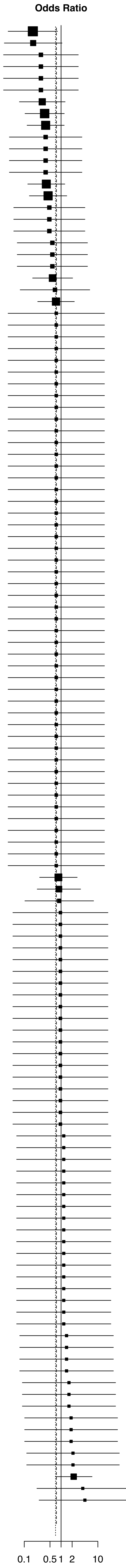

Supplement: Suppl. Figure 2. Forest plot showing the enrichments for altered genes in patients with Ki67 21-55% relative to those with Ki67>55% (illustrated as odds ratio [OR] where OR<1 indicates enrichment in patients with Ki67 21-55% and OR>1 indicates enrichment in patients with Ki67>55%). The plot incudes  [file supplementary_figure_2.pdf]
