# Supplementary material for: The molecular characteristics of high-grade gastroenteropancreatic neuroendocrine neoplasms
Source: Endocr Relat Cancer. 2021 Oct 14;29(1):1–14. doi: 10.1530/ERC-21-0152 (PMC8630776; doi:10.1530/ERC-21-0152)

Study	OR	95%-CI	Weight (random)
NTRK3	0.07	[0.00; 1.26]	0.3%
SMAD4	0.08	[0.00; 1.46]	0.3%
AR	0.11	[0.01; 2.11]	0.3%
CDH2	0.11	[0.01; 2.11]	0.3%
MAP3K4	0.11	[0.01; 2.11]	0.3%
PTCH1	0.11	[0.01; 2.11]	0.3%
CIC	0.14	[0.01; 2.68]	0.3%
EMSY	0.14	[0.01; 2.68]	0.3%
MAP4K4	0.14	[0.01; 2.68]	0.3%
MITF	0.14	[0.01; 2.68]	0.3%
PDGFRA	0.14	[0.01; 2.68]	0.3%
KMT2A	0.15	[0.02; 1.27]	0.5%
RNF43	0.15	[0.02; 1.27]	0.5%
ACVR2A	0.18	[0.01; 3.63]	0.3%
AXIN1	0.18	[0.01; 3.63]	0.3%
BCOR	0.18	[0.01; 3.63]	0.3%
EPHA6	0.18	[0.01; 3.63]	0.3%
ERCC3	0.18	[0.01; 3.63]	0.3%
ERCC4	0.18	[0.01; 3.63]	0.3%
FGFR4	0.18	[0.01; 3.63]	0.3%
GATA3	0.18	[0.01; 3.63]	0.3%
JAK3	0.18	[0.01; 3.63]	0.3%
LTK	0.18	[0.01; 3.63]	0.3%
NGFR	0.18	[0.01; 3.63]	0.3%
NKX2-1	0.18	[0.01; 3.63]	0.3%
NOTCH2	0.18	[0.01; 3.63]	0.3%
PIK3R1	0.18	[0.01; 3.63]	0.3%
PTEN	0.18	[0.01; 3.63]	0.3%
RUNX1	0.18	[0.01; 3.63]	0.3%
SETD2	0.18	[0.01; 3.63]	0.3%
SF3B1	0.18	[0.01; 3.63]	0.3%
TGFBR2	0.18	[0.01; 3.63]	0.3%
WT1	0.18	[0.01; 3.63]	0.3%
BRAF	0.21	[0.07; 0.58]	2.3%
NOTCH1	0.21	[0.02; 1.80]	0.5%
RICTOR	0.26	[0.03; 2.25]	0.5%
CDKN2A	0.32	[0.04; 2.97]	0.5%
ERBB2	0.32	[0.04; 2.97]	0.5%
FLT4	0.32	[0.04; 2.97]	0.5%
GPC5	0.32	[0.04; 2.97]	0.5%
IGF2R	0.32	[0.04; 2.97]	0.5%
KDM6A	0.32	[0.04; 2.97]	0.5%
NOTCH3	0.32	[0.04; 2.97]	0.5%
NOTCH4	0.32	[0.04; 2.97]	0.5%
MYC	0.36	[0.18; 0.70]	5.4%
ABL1	0.44	[0.04; 4.30]	0.5%
ARID1B	0.44	[0.04; 4.30]	0.5%
ASXL1	0.44	[0.04; 4.30]	0.5%
BPTF	0.44	[0.04; 4.30]	0.5%
BRCA1	0.44	[0.04; 4.30]	0.5%
DICER1	0.44	[0.04; 4.30]	0.5%
EPHA7	0.44	[0.04; 4.30]	0.5%
FOXO1	0.44	[0.04; 4.30]	0.5%
KEAP1	0.44	[0.04; 4.30]	0.5%
KIT	0.44	[0.04; 4.30]	0.5%
MED12	0.44	[0.04; 4.30]	0.5%
MET	0.44	[0.04; 4.30]	0.5%
TNFAIP3	0.44	[0.04; 4.30]	0.5%
ARID1A	0.46	[0.23; 0.92]	5.2%
ESR1	0.48	[0.22; 1.03]	4.0%
ATRX	0.52	[0.10; 2.77]	0.9%
EP400	0.52	[0.10; 2.77]	0.9%
EPHB1	0.52	[0.10; 2.77]	0.9%
PIK3CA	0.52	[0.10; 2.77]	0.9%
SLIT2	0.52	[0.10; 2.77]	0.9%
CTNNB1	0.65	[0.16; 2.72]	1.2%
BRCA2	0.66	[0.12; 3.71]	0.8%
SMO	0.66	[0.12; 3.71]	0.8%
TSC2	0.66	[0.12; 3.71]	0.8%
TP53	0.67	[0.34; 1.30]	5.4%
KRAS	0.79	[0.36; 1.72]	3.9%
SMARCA4	0.79	[0.18; 3.45]	1.1%
SOX9	0.79	[0.18; 3.45]	1.1%
GNAS	0.88	[0.30; 2.61]	2.0%
EPHA3	0.89	[0.14; 5.48]	0.7%
ERBB4	0.89	[0.14; 5.48]	0.7%
FANCA	0.89	[0.14; 5.48]	0.7%
MAP2K4	0.89	[0.14; 5.48]	0.7%
MYO3A	0.89	[0.14; 5.48]	0.7%
PIK3CG	0.89	[0.14; 5.48]	0.7%
RET	0.89	[0.14; 5.48]	0.7%
ROS1	0.89	[0.14; 5.48]	0.7%
APC	0.95	[0.46; 1.94]	4.7%
ATM	0.97	[0.49; 1.91]	5.2%
CHD1	1.00	[0.22; 4.65]	1.0%
KDM5A	1.07	[0.56; 2.04]	5.8%
PREX2	1.08	[0.28; 4.17]	1.3%
KMT2D	1.08	[0.40; 2.91]	2.4%
AMER1	1.35	[0.26; 6.94]	0.9%
PRDM9	1.35	[0.26; 6.94]	0.9%
TP73	1.35	[0.26; 6.94]	0.9%
NF1	1.37	[0.38; 4.93]	1.5%
RB1	1.61	[0.84; 3.08]	5.7%
FBXW7	1.80	[0.67; 4.84]	2.4%
EP300	2.06	[0.33; 12.68]	0.7%
PLCG1	2.06	[0.33; 12.68]	0.7%
KMT2C	2.79	[0.49; 15.70]	0.8%
MAP3K14	9.80	[0.50; 193.12]	0.3%
MAP3K1	12.80	[0.68; 242.19]	0.3%

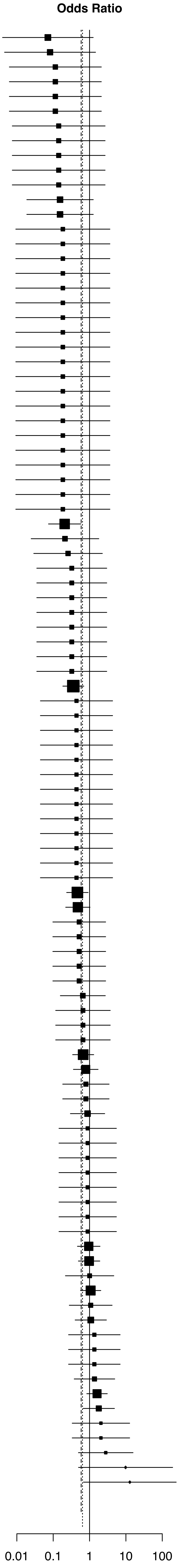

Supplement: Suppl. Figure 11. Forest plot showing the enrichments for altered genes in patients with the large cell relative to the small cell NEC (illustrated as odds ratio [OR] where OR<1 indicates enrichment in large cell NEC and OR>1 indicates enrichment in small cell NEC). The plot incudes all genes mutate [file supplementary_figure_11.pdf]
