# Supplementary material for: The molecular characteristics of high-grade gastroenteropancreatic neuroendocrine neoplasms
Source: Endocr Relat Cancer. 2021 Oct 14;29(1):1–14. doi: 10.1530/ERC-21-0152 (PMC8630776; doi:10.1530/ERC-21-0152)
Supplement: Suppl.Figure 12. Oncoplot showing the top 50 most frequently altered genes (rows) among 29 NET–G3 patients (columns). Upper panel shows the mutational burden per sample. Percentages on the right represent mutations frequency per gene. The panel under the oncoplot area is composed of one single row h [file supplementary_figure_12.pdf]

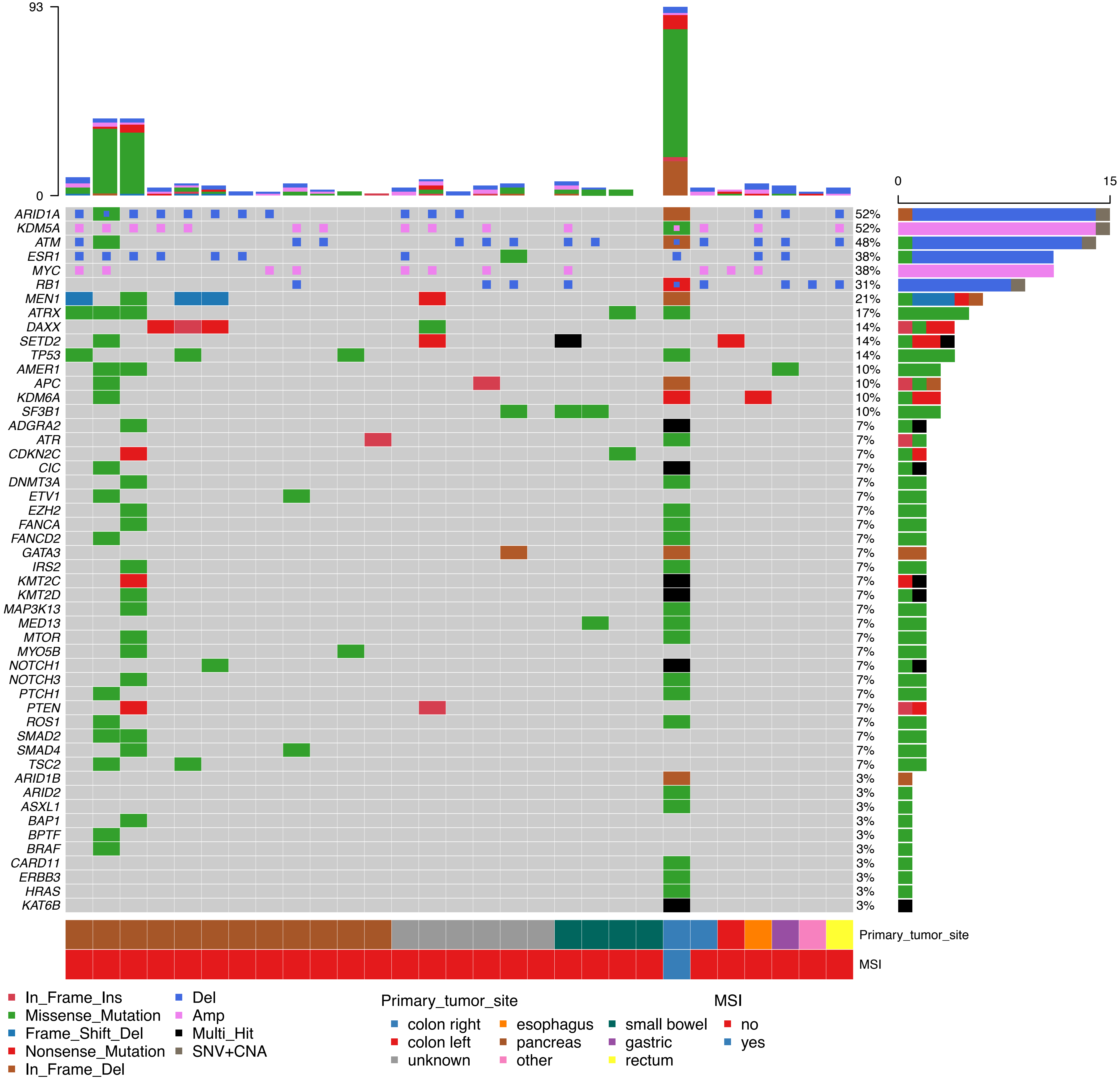

93
0
15
ARID1A 52%
KDM5A 52%
ATM 48%
ESR1 38%
MYC 38%
RB1 31%
MEN1 21%
ATRX 17%
DAXX 14%
SETD2 14%
TP53 14%
AMER1 10%
APC 10%
KDM6A 10%
SF3B1 10%
ADGRA2 7%
ATR 7%
CDKN2C 7%
CIC 7%
DNMT3A 7%
ETV1 7%
EZH2 7%
FANCA 7%
FANCD2 7%
GATA3 7%
IRS2 7%
KMT2C 7%
KMT2D 7%
MAP3K13 7%
MED13 7%
MTOR 7%
MYO5B 7%
NOTCH1 7%
NOTCH3 7%
PTCH1 7%
PTEN 7%
ROS1 7%
SMAD2 7%
SMAD4 7%
TSC2 7%
ARID1B 3%
ARID2 3%
ASXL1 3%
BAP1 3%
BPTF 3%
BRAF 3%
CARD11 3%
ERBB3 3%
HRAS 3%
KAT6B 3%
Primary_tumor_site
MSI
In_Frame_Ins
Missense_Mutation
Frame_Shift_Del
Nonsense_Mutation
In_Frame_Del
Del
Amp
Multi_Hit
SNV+CNA
Primary_tumor_site
colon right
colon left
unknown
esophagus
pancreas
other
small bowel
gastric
rectum
MSI
no
yes
