# Supplementary material for: The molecular characteristics of high-grade gastroenteropancreatic neuroendocrine neoplasms
Source: Endocr Relat Cancer. 2021 Oct 14;29(1):1–14. doi: 10.1530/ERC-21-0152 (PMC8630776; doi:10.1530/ERC-21-0152)

Study	OR	95%-CI	Weight (random)
KRAS	0.05	[0.00; 0.89]	0.5%
TP53	0.08	[0.02; 0.24]	2.9%
BRAF	0.09	[0.01; 0.69]	0.9%
FBXW7	0.16	[0.01; 2.83]	0.5%
KMT2A	0.16	[0.01; 2.83]	0.5%
AR	0.25	[0.01; 4.74]	0.4%
RICTOR	0.25	[0.01; 4.74]	0.4%
SLIT2	0.25	[0.01; 4.74]	0.4%
SMARCA4	0.25	[0.01; 4.74]	0.4%
SOX9	0.25	[0.01; 4.74]	0.4%
APC	0.29	[0.08; 1.03]	2.3%
GNAS	0.31	[0.04; 2.55]	0.9%
BRCA2	0.31	[0.02; 6.02]	0.4%
EMSY	0.31	[0.02; 6.02]	0.4%
ERBB2	0.31	[0.02; 6.02]	0.4%
FLT4	0.31	[0.02; 6.02]	0.4%
GPC5	0.31	[0.02; 6.02]	0.4%
IGF2R	0.31	[0.02; 6.02]	0.4%
MAP4K4	0.31	[0.02; 6.02]	0.4%
MITF	0.31	[0.02; 6.02]	0.4%
SMO	0.31	[0.02; 6.02]	0.4%
NTRK3	0.35	[0.04; 2.95]	0.8%
RNF43	0.35	[0.04; 2.95]	0.8%
MYC	0.37	[0.16; 0.89]	5.1%
ABL1	0.41	[0.02; 8.16]	0.4%
ACVR2A	0.41	[0.02; 8.16]	0.4%
AXIN1	0.41	[0.02; 8.16]	0.4%
BCOR	0.41	[0.02; 8.16]	0.4%
EPHA3	0.41	[0.02; 8.16]	0.4%
EPHA6	0.41	[0.02; 8.16]	0.4%
EPHA7	0.41	[0.02; 8.16]	0.4%
ERBB4	0.41	[0.02; 8.16]	0.4%
ERCC3	0.41	[0.02; 8.16]	0.4%
ERCC4	0.41	[0.02; 8.16]	0.4%
FGFR4	0.41	[0.02; 8.16]	0.4%
FOXO1	0.41	[0.02; 8.16]	0.4%
KIT	0.41	[0.02; 8.16]	0.4%
LTK	0.41	[0.02; 8.16]	0.4%
MED12	0.41	[0.02; 8.16]	0.4%
MET	0.41	[0.02; 8.16]	0.4%
MYO3A	0.41	[0.02; 8.16]	0.4%
NKX2-1	0.41	[0.02; 8.16]	0.4%
NOTCH2	0.41	[0.02; 8.16]	0.4%
PIK3CG	0.41	[0.02; 8.16]	0.4%
PIK3R1	0.41	[0.02; 8.16]	0.4%
PRDM9	0.41	[0.02; 8.16]	0.4%
RUNX1	0.41	[0.02; 8.16]	0.4%
TGFBR2	0.41	[0.02; 8.16]	0.4%
TNFAIP3	0.41	[0.02; 8.16]	0.4%
WT1	0.41	[0.02; 8.16]	0.4%
CTNNB1	0.48	[0.06; 4.18]	0.8%
KMT2D	0.57	[0.12; 2.77]	1.5%
CDH2	0.59	[0.07; 5.23]	0.8%
EP400	0.59	[0.07; 5.23]	0.8%
EPHB1	0.59	[0.07; 5.23]	0.8%
MAP3K4	0.59	[0.07; 5.23]	0.8%
NF1	0.59	[0.07; 5.23]	0.8%
PIK3CA	0.59	[0.07; 5.23]	0.8%
PREX2	0.59	[0.07; 5.23]	0.8%
RB1	0.70	[0.29; 1.72]	4.8%
CDKN2A	0.74	[0.08; 6.91]	0.8%
CHD1	0.74	[0.08; 6.91]	0.8%
NOTCH4	0.74	[0.08; 6.91]	0.8%
PDGFRA	0.74	[0.08; 6.91]	0.8%
SMAD4	0.85	[0.17; 4.32]	1.4%
ARID1B	1.00	[0.10; 10.01]	0.7%
ASXL1	1.00	[0.10; 10.01]	0.7%
BPTF	1.00	[0.10; 10.01]	0.7%
BRCA1	1.00	[0.10; 10.01]	0.7%
DICER1	1.00	[0.10; 10.01]	0.7%
JAK3	1.00	[0.10; 10.01]	0.7%
KEAP1	1.00	[0.10; 10.01]	0.7%
MAP2K4	1.00	[0.10; 10.01]	0.7%
NGFR	1.00	[0.10; 10.01]	0.7%
NOTCH1	1.00	[0.19; 5.25]	1.4%
RET	1.00	[0.10; 10.01]	0.7%
TP73	1.00	[0.10; 10.01]	0.7%
ARID1A	1.20	[0.52; 2.79]	5.4%
PTCH1	1.21	[0.22; 6.63]	1.3%
KDM5A	1.26	[0.54; 2.92]	5.4%
ESR1	1.29	[0.54; 3.09]	5.0%
CIC	1.54	[0.27; 8.86]	1.2%
NOTCH3	1.54	[0.27; 8.86]	1.2%
TSC2	1.54	[0.27; 8.86]	1.2%
ATM	1.77	[0.76; 4.16]	5.3%
FANCA	2.07	[0.33; 13.07]	1.1%
GATA3	2.07	[0.33; 13.07]	1.1%
PTEN	2.07	[0.33; 13.07]	1.1%
ROS1	2.07	[0.33; 13.07]	1.1%
KDM6A	2.39	[0.50; 11.40]	1.6%
AMER1	3.23	[0.61; 16.99]	1.4%
SF3B1	3.23	[0.61; 16.99]	1.4%
ATRX	3.42	[0.91; 12.80]	2.2%
SETD2	4.48	[0.94; 21.36]	1.6%
MEN1	11.09	[2.10; 58.62]	1.4%
DAXX	13.76	[1.47; 128.75]	0.8%

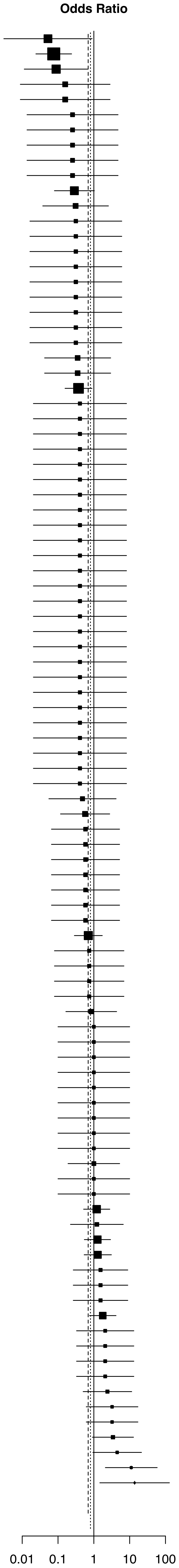

Supplement: Suppl. Figure 14. Forest plot showing the enrichments for altered in patients with large cell NEC versus NET G3 (illustrated as odds ratio [OR] where OR<1 indicates enrichment in large cell NEC and OR>1 indicates enrichment in NET G3). The plot incudes all genes mutated in minimum of 3 of the patien [file supplementary_figure_14.pdf]
